# On the way toward regulatable expression systems in acetic acid bacteria: target gene expression and use cases

**DOI:** 10.1007/s00253-021-11269-z

**Published:** 2021-04-15

**Authors:** Philipp Moritz Fricke, Angelika Klemm, Michael Bott, Tino Polen

**Affiliations:** IBG-1: Biotechnology, Institute of Bio- and Geosciences, Forschungszentrum Jülich GmbH, 52425 Jülich, Germany

**Keywords:** Acetic acid bacteria, *Acetobacteraceae*, Plasmid, Origin, Promoter, Induction

## Abstract

**Abstract:**

Acetic acid bacteria (AAB) are valuable biocatalysts for which there is growing interest in understanding their basics including physiology and biochemistry. This is accompanied by growing demands for metabolic engineering of AAB to take advantage of their properties and to improve their biomanufacturing efficiencies. Controlled expression of target genes is key to fundamental and applied microbiological research. In order to get an overview of expression systems and their applications in AAB, we carried out a comprehensive literature search using the Web of Science Core Collection database. The *Acetobacteraceae* family currently comprises 49 genera. We found overall 6097 publications related to one or more AAB genera since 1973, when the first successful recombinant DNA experiments in *Escherichia coli* have been published. The use of plasmids in AAB began in 1985 and till today was reported for only nine out of the 49 AAB genera currently described. We found at least five major expression plasmid lineages and a multitude of further expression plasmids, almost all enabling only constitutive target gene expression. Only recently, two regulatable expression systems became available for AAB, an *N*-acyl homoserine lactone (AHL)-inducible system for *Komagataeibacter rhaeticus* and an l-arabinose-inducible system for *Gluconobacter oxydans*. Thus, after 35 years of constitutive target gene expression in AAB, we now have the first regulatable expression systems for AAB in hand and further regulatable expression systems for AAB can be expected.

**Key points:**

• *Literature search revealed developments and usage of expression systems in AAB.*

• *Only recently 2 regulatable plasmid systems became available for only 2 AAB genera.*

• *Further regulatable expression systems for AAB are in sight.*

## Introduction

Acetic acid bacteria (AAB) are a group of obligately aerobic Gram-negative bacteria that exhibit a unique form of metabolism by which they typically partially oxidize a variety of substrates such as sugars or ethanol by membrane-bound dehydrogenases (mDHs) and produce acetic acid. AAB are already used for a long time and they are very important for the production of foods and beverages such as vinegar, kombucha, kefir, and other products. Several reviews on AAB are already available that provide overviews, discussions and outlooks on issues related to their taxonomy, physiology and biochemistry, fermentation (foods and beverages), acetic acid resistance, production of exopolysaccharides including bacterial cellulose, pyrroloquinoline quinone (PQQ)-dependent dehydrogenases, oxidation of carbon sources and alcohols, nitrogen fixation, metabolic engineering, biotechnological and industrial applications, as well as their use as microbial biosensors (see, for example, Cleenwerck and De Vos 2008; De Roos and De Vuyst 2018; Gao et al. 2020b; Gomes et al. 2018; Gullo et al. 2018; Kolesovs and Semjonovs 2020; La China et al. 2018; Laureys et al. 2020; Lynch et al. 2019; Mamlouk and Gullo 2013; Matsutani and Yakushi 2018; Trcek et al. 2015; Wang et al. 2015; Zhang et al. 2020). No review is currently available that provides an overview of the expression systems developed, tested and used for target gene expression and their use cases in AAB.

Generally, the expression of target genes to produce proteins in bacterial cell culture for various purposes is a standard method in basic research and biotechnological applications. For constitutive and inducible or regulatable expression, numerous plasmids have already been developed and established in many bacteria, which have been summarized and reviewed in the past for bacteria other than AAB (see, for example, Chen 2012; Connell 2001; Dilworth et al. 2018; Evans and Mizrahi 2015; Forstner et al. 2007; Gruber et al. 2015; Parachin et al. 2012; Schnappinger and Ehrt 2014; Terpe 2006; Valero 2012). In AAB, for a long time, only constitutive target gene expression and in a very few studies only weakly regulatable target gene expression was achieved since, for example, the transfer of heterologous regulatable systems such as the well-known classical examples of the TetR-, AraC-, and LacI-dependent systems and their application was not really successful yet in AAB due to high (leaky) expression already in the absence of the respective inducer (Florea et al. 2016a; Teh et al. 2019). Only two regulatable expression systems, an *N*-acyl homoserine lactone-inducible *luxR*-P_*lux*_ system for *Komagataeibacter rhaeticus* and an l-arabinose-inducible *araC*-P_*araBAD*_ system for *Gluconobacter oxydans* with up to 480-fold induction have recently been reported for the two AAB species (Florea et al. 2016a; Fricke et al. 2020). To provide a comprehensive overview of expression systems, their latest developments and the use cases in AAB we have searched and evaluated the literature for relevant AAB studies.

## Literature search and updates in the systematics of AAB genera

We carried out a keyword-based literature search using the Web of Science Core Collection (WoSCC) database. Currently, for the *Acetobacteraceae* family there are 49 genera listed in the taxonomy browser of the NCBI website. We assumed that a publication potentially relevant for finding expression plasmids in AAB should contain at least the name of one of these AAB genera in the title, abstract, or keywords, respectively (for *Stella* we used the two full names *Stella humosa* and *Stella vacuolata*). In this first literature filtering step, the single AAB genus word-based searches in the WoSCC database revealed that the top 3 AAB genera with by far the most publications were *Acetobacter* (3,774), *Gluconobacter* (1,446) and *Glucon(o)acetobacter* (1,194), respectively. These top 3 genera were followed by *Acidiphilium* (345), *Komagataeibacter* (229), *Roseomonas* (177), *Asaia* (175), *Acidocella* (62) and *Acidomonas* (33). As outlined below, some genera names were introduced only recently and many of the included species had a different genus name before. Together, these 9 AAB genera already represent 97.6% (6,288) of all (6,440) non-redundant publications somehow related to one or more out of the 49 AAB genera. We then selected the 6097 publications from the year 2020 backward to 1973, the year of the first published successful DNA cloning experiments in *Escherichia coli* (Cohen et al. 1973). Using EndNote, for 3397 publications (55.7%), we could directly get access to the full text (PDF) due to Open Access or journal access via our institutions’ central library. To narrow down the AAB-related publication list in a second step, we filtered title, abstract, keywords, and, if available, the full text (PDF) on the one hand by checking for the presence of the text strings *plasmid*, *vector*, *host*, *induction*, *inducer*, *activation*, *activator*, *repression*, *repressor*, *expression*, *promoter*, *regulation* or *terminator*, respectively. On the other hand, we also checked the publications by manual inspection of title, abstract and, if available, full-text PDF in regard to expression systems, the genetic work and the case studies. We finally ended up with a list of 243 publications from 1985 onward representing studies in which plasmids have been created, tested or used in 9 AAB genera. We probably missed a number of studies reporting plasmids for expression in AAB due to missing full-text information.

**Fig. 1:**
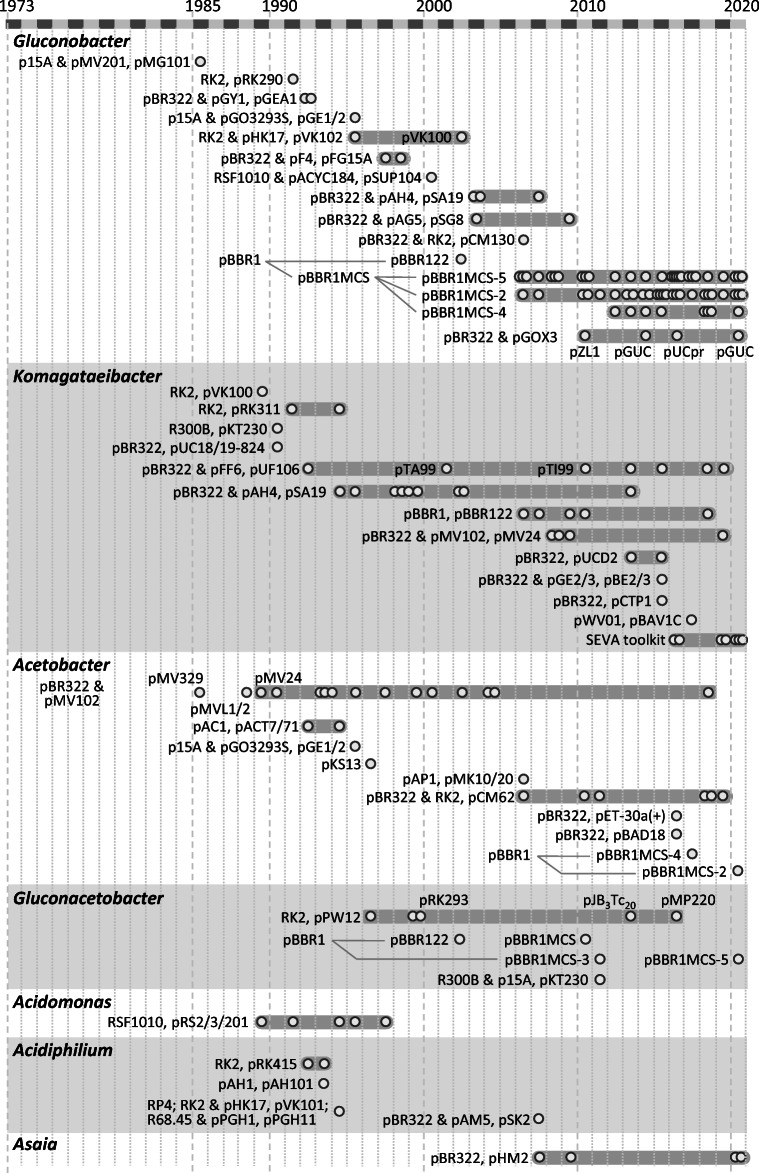
Time plot illustrating major lineages and diversity of expression plasmids and their origins of replication hitherto used in AAB and found for seven out of 49 AAB genera according to the publications (o) in the WoSCC database

It should be mentioned that the taxonomy of the AAB has gone through some updates including the creation of new AAB genera and renaming of AAB species already described in the literature. In 1989 the genus *Acidomonas* was proposed to include a group of acidophilic, facultatively methylotrophic bacteria (Urakami et al. 1989). These microorganisms are Gram-negative, non-spore forming, non-motile, and rod-shaped and grow at pH 2.0 to 5.5. These characteristics are unique among the methanol-utilizing bacteria and the typical strain *Acetobacter methanolicus* can be distinguished from the type and representative strains of *Acetobacter*, *Gluconobacter*, and *Acidiphilium*, resulting in the renaming of *Acetobacter methanolicus* to *Acidomonas methanolica*. In 1997 a genus concept of AAB was proposed classifying *Acetobacter*, *Gluconoacetobacter*, *Acidomonas*, and *Gluconobacter* based on partial 16S rRNA sequences (Yamada et al. 1997). In 1998 *Gluconoacetobacter* was changed to *Gluconacetobacter* (International Committee on Systematic Bacteriology). As a consequence of these changes and updates, *Acetobacter diazotrophicus*, *A. europaeus*, *A. hansenii*, *A. liquefaciens*, and *A. xylinum* (alias *A. xylinus*) were included in the genus *Gluconacetobacter* with the respective changes in nomenclature: *Gluconacetobacter diazotrophicus*, *Ga. europaeus*, *Ga. hansenii*, *Ga. liquefaciens*, and *Ga. xylinus*, respectively. Furthermore, in the following years, a phylogenetic duality in the new genus *Gluconacetobacter* was found. The *Ga. liquefaciens* group and the *Ga. xylinus* group could be phylogenetically, phenotypically and ecologically distinguished from each other at the generic level. This resulted in the creation of the new AAB genus *Komagataeibacter* based on 16S rRNA gene sequences and the transfer of several new combinations including *Ga. xylinus* (formerly *A. xylinus* alias *A. xylinum*) to *Komagataeibacter xylinus* on the basis of taxonomic characteristics (Yamada 2014; Yamada et al. 2012). Taking these AAB genera updates and renaming of AAB species into account for the publications before 1989, 1998, and 2014 (and after), we found in our final list 101 publications related to *Gluconobacter*, 61 to *Komagataeibacter*, 41 to *Acetobacter*, 20 to *Gluconacetobacter*, 8 to *Acidiphilium*, 6 to *Acidomonas*, 4 to *Asaia*, 1 to *Kozakia*, and 1 to *Roseomonas*, respectively. Among these 243 publications, in 59 studies genomic allele replacements and transposon- or plasmid-based gene inactivation screenings requiring the functional expression of transposases or resistance genes as well as homologous recombination were carried out using a few strategies without further recombinant target gene expression. These studies were excluded from our review which removed the genera *Kozakia* and *Roseomonas* from our list. The remaining 184 publications were organized according to the expression plasmids used, the case studies or their timelines, and are presented for the seven remaining AAB genera according to the number of publications (Fig. 1).

## Target gene expression in *Gluconobacter*

Most studies reporting on the construction and usage of expression plasmids in AAB were found for the genus *Gluconobacter* (81). Initially, in 11 studies, several plasmids were constructed and tested before a major expression plasmid family was established in *Gluconobacter* in 2006.

### Plasmid diversity before establishing the major expression plasmid lineage

The first study reporting an *E. coli* / *Gluconobacter* shuttle vector was published in 1985 by Teruhiko Beppu and co-workers, who in parallel were also working on shuttle vectors for *Acetobacter* (see *Acetobacter* section). This first shuttle vector for *Gluconobacter* was constructed by ligation of cryptic plasmid pMV201 (2.5 kb) found in *G. suboxydans* IFO 3130 with the *E. coli* plasmid pACYC177 (3.9 kb) carrying the p15A *ori*. The resulting chimeric plasmid pMG101 (6.2 kb) carries an ampicillin (Amp) resistance gene and replicates in *G. suboxydans* as well as in *E. coli* (Fukaya et al. 1985b). Later, conjugal transfer of a series of plasmids of the incompatibility group P (RP4, RP1::Tn*951*, pRK290 and derivatives thereof) and Q (pKT230/231) was studied. A gentamycin (Gm) resistance-encoding pRK290 derivative (20 kb, minimal replicon derivative of RK2) was constructed and suggested to offer considerable potential as a versatile gene delivery system for *Gluconobacter* (Condon et al. 1991). The promoter of the Gm resistance gene is functional since pRK290::Gm increased the minimal inhibitory concentration for gentamycin from 80 μg/mL to more than 2 mg/mL. With plasmid RP1::Tn*951* carrying the lactose transposon, heterologous gene expression and regulation was tested in *Gluconobacter*. Tn*951* contains IPTG-inducible and cAMP receptor protein (CRP)-dependent genes homologous to the *E. coli lacI*, *lacZ*, and *lacY* genes. The Tn*951*-based β-galactosidase activity was found to be induced 4-fold to 6-fold by IPTG in *Gluconobacter*, which suggested the synthesis of a functional LacI repressor in *Gluconobacter*, yet the repression was leaky in the absence of IPTG and the induced LacZ activity less than 5% compared to fully induced levels in *E. coli*. Since in *E. coli* the *lac* operon displays reversible cAMP-dependent catabolite repression with glucose, cAMP was exogenously added (100 μM) to the *Gluconobacter* culture. cAMP did not result in any detectable increase in LacZ activity, suggesting that a catabolite repression-like phenomenon was unlikely to be the cause of the poor LacZ activity in *Gluconobacter* if the assumption holds true that cAMP can be transported into the cell at levels sufficient for physiological effects to be observed. With the genome sequences available today we know that a CRP gene appears to be absent in *Gluconobacter*. The low levels of LacZ activity could be explained by inefficient recognition of the heterologous Tn*951* promoter in *Gluconobacter* (Condon et al. 1991).

Generally, RK2-based vectors such as the broad host range vector pRK293 (21.4 kb) can be transferred to *G. oxydans* by bacterial mating, yet efforts to use this cloning vehicle were unsuccessful, since the transfer of pRK293 occurred only with very low efficiency and with pRK293 containing a 4.7 kb insert of interest no clones could be obtained (Cleton-Jansen et al. 1991). In another attempt to construct and test a suitable shuttle vector, the small cryptic plasmid pGY1 (2.7 kb) also detected in *G. suboxydans* IFO 3130 was cleaved and ligated with cleaved pUC18 (Takeda and Shimizu 1992). The resulting chimeric vector pGEA1 (5.4 kb) carries ampicillin resistance and was used to express the cytochrome *c*-553 gene of *G. suboxydans* IFO 12528 from its native promoter region in *G. suboxydans* IFO 3254, which oxidizes ethanol poorly because of a deficiency of the second subunit of the membrane-bound alcohol dehydrogenase. Transformants of IFO 3254 exhibited ethanol oxidation activity and increased dehydrogenase activities for d-glucose, d-sorbitol and glycerol. Furthermore, azide insensitivity of the respiratory chain was restored, which indicated that cytochrome *c*-553 is in the pathway of the azide-insensitive respiratory chain bypass of *G. suboxydans* (Takeda and Shimizu 1992; Takeda et al. 1992). While in these early *Gluconobacter* studies chemically competent cells or conjugation were used to transfer plasmids, electroporation was also established for *Gluconobacter* and enabled transformation frequencies of up to 10^5^ transformants /μg of DNA (Creaven et al. 1994). In the following, conjugation and electroporation were used for *Gluconobacter* almost equally often.

In a further attempt to construct, test, and establish a suitable shuttle vector, the chimeric plasmid pGE1 (11.9 kb) was constructed from the endogenous cryptic plasmid pGO3293S (9.9 kb) found in *G. oxydans* IFO 3293 and plasmid pSUP301 (5 kb) from *E. coli* (Shinjoh and Hoshino 1995). The plasmid pGO3293S encodes enzyme activities that converts l-sorbose to 2-keto-l-gulonic acid, the non-lactonized precursor of vitamin C. Plasmid pSUP301 contains pACYC177 introduced above. The plasmid pGE1 could be transferred into *G. oxydans* IFO 3293 with a high frequency (10^-1^ transconjugants per recipient) by conjugal transfer, maintained very stably without antibiotic selection, and did not inhibit the growth or 2-keto-l-gulonic acid productivity of producer strains derived from *G. oxydans* IFO 3293. pGE1 could also be shortened to a 9.8-kb plasmid termed pGE2. pGE1 could be introduced in 6 *Gluconobacter* and 4 *Acetobacter* strains out of 6 and 28 strains tested. Thus, it showed a broader host-range than the shuttle vector pMG101 introduced above, which showed a limited host-range even in *Gluconobacter*. pGE1 was considerably stable in the transconjugants after two cycles of cultivation without kanamycin, except in *G. frateurii* IFO 3271. The usefulness of pGE1 as an expression vector was confirmed by subcloning the membrane-bound l-sorbosone dehydrogenase (SNDH) gene of *A. liquefaciens* IFO 12258 and its expression in *G. oxydans* IFO 3293 derivatives. Using resting cells, pGE1 derivatives with the SNDH gene, however, did not lead to a higher 2-keto-l-gulonic acid production than the pVK102 derivative with the SNDH gene (Shinjoh and Hoshino 1995). Already beforehand, plasmid pVK102 (23 kb), a mobilizable broad-host-range cosmid vector constructed from pRK290 and pHK17 via the intermediate pVK100 originally used in *Agrobacterium* and providing kanamycin and tetracycline resistance, was used to construct a genomic library of *A. liquefaciens* IFO 12258 for a SNDH activity screening in a *G. oxydans* IFO 3293 mutant that accumulates l-sorbosone in the presence of l-sorbose (Shinjoh et al. 1995). Plasmid pVK100 was used in a check of the biological functions of the polyol dehydrogenase genes *sldAB* to complement mutants of *G. oxydans* DSM 4025 disrupted in the *sldAB* genes by expressing *sldA*, *sldB*, or both genes from *G. oxydans* promoter P_*A*_ (Shinjoh et al. 2002).

In another attempt to create a shuttle vector, the endogenous plasmid pF4 (4.4 kb) extracted from *G. oxydans* T-100 was ligated with plasmid pHSG298 (2.7 kb) carrying pBR322 *ori* and the resulting chimeric plasmid pFG15A (7.3 kb) was confirmed to be also stable in *G. oxydans* G624 across several passages in a broth without kanamycin and had no effects on the l-sorbose production by the host (Saito et al. 1997). Plasmid pFG15A was used to express l-sorbose and l-sorbosone dehydrogenase genes of *G. oxydans* T-100 from their native promoter regions. *G. oxydans* G624, which is unable to produce 2-keto-l-gulonic acid endogenously, cultivated in the presence of d-sorbitol and with the expression plasmid reached a 2-keto-l-gulonic acid titer 2.3-fold higher than the titer obtained by *G. oxydans* T-100, yet the yield was still insufficient (Saito et al. 1997). Therefore, to improve the expression of the dehydrogenase genes, the native promoter region was replaced with that of *E. coli* P_*tufB1*_, P_*lac*_, or P_L_, which all were active. In combination with chemical mutagenesis of the strain, the 2-keto-l-gulonic acid production level was further increased 3-fold (Saito et al. 1998).

In another study, the broad-host-range vector pSUP104, which includes the mobilization and replication functions of the IncQ plasmid RSF1010 and was originally tested in *Rhizobium*, *Agrobacterium*, and *Pseudomonas* species, respectively, was used in *Gluconobacter* to complement a Tn*5*-based *pqqE*-deficient mutant by expressing a genomic library fragment containing the *pqqE* gene of the PQQ coenzyme biosynthesis gene cluster *pqqBCDE* of *G. oxydans* ATCC 9937 (Felder et al. 2000). RSF1010 was used otherwise in some *Acidomonas* studies and in an *ori* test using the pSEVA tool kit in *Komagataeibacter* (see sections below).

Another expression plasmid used in *Gluconobacter* is the shuttle vector pSA19 already developed and introduced for use in *Komagataeibacter* in 1994 (see *Komagataeibacter* section). The feasibility of using pSA19 was also demonstrated for *Gluconobacter* by expressing the xylitol dehydrogenase (XDH) gene of *Morganella morganii* from the promoter P_*lac*_ (Tonouchi et al. 2003). In the recombinant *G. oxydans* ATCC 621 strain, the XDH activity was increased 4-fold. To develop an improved production process for the alternative sweetener xylitol, also the endogenous XDH gene from *G. oxydans* ATCC 621 was cloned with and without its native promoter region into pSA19 (Sugiyama et al. 2003). Expression from P_*lac*_ alone increased the XDH activity 5-fold in the recombinant *G. oxydans* strain, while XDH activity was 11-fold higher when the gene was expressed from both P_*lac*_ and its native promoter. The latter construct increased the xylitol titer obtained by conversion of d-arabitol to xylulose with arabitol dehydrogenase followed by reduction to xylitol 2-fold within 48 h when ethanol was added. Later pSA19 was used to express the *sboA* and *sboR* genes both located upstream of the FAD-dependent d-sorbitol dehydrogenase (SLDH) genes *sldSLC* in *G. frateurii* THD32 to analyze SboA enzyme activity and the role of the putative transcriptional regulator SboR in the regulation of *sldSLC* expression (Soemphol et al. 2007). The SboA enzyme showed NADPH-dependent l-sorbose reductase activity. The predicted transcriptional regulator SboR was suggested to be a repressor of *sboA* expression while another regulator was assumed to be required for the induction of *sldSLC* by d-sorbitol or l-sorbose. When *sboR* was disrupted in *G. frateurii*, the SboA activity was increased 1.5-fold and 2-fold when grown on d-sorbitol and l-sorbose, respectively. Since the basal expression in the parental strain was already relatively high according to the activities in the cytosolic fraction (0.6 to 0.9 U/mg) the SboR-repressed promoter of the *sboRA* operon seems not suitable to be used for a regulatable expression system in *Gluconobacter*.

In parallel to the test and usage of pSA19 in *Gluconobacter*, another shuttle plasmid was created and tested. The cryptic endogenous plasmid pAG5 (5.6 kb) found in *G. oxydans* IFO 3171 was ligated with pUC18 and the chimeric vector was termed pSG8 (Tonouchi et al. 2003). Additionally, the 2 kb smaller plasmid pSG6 was created by *Eco*RI digestion of pSG8 and self-ligation of the 3.6 kb fragment. The copy numbers of pSG8/6 were estimated to be 10 per genome and both were found to be stable in *G. oxydans* ATCC 621 after 10 days of repeated cultivation (50 generations) in the absence of ampicillin. Plasmid pSG6 was used for expression of the endogenous cyanide-insensitive quinol oxidase genes *cioAB* in the wild type *G. suboxydans* IFO 12528 (Mogi et al. 2009). Additional plasmid-based expression was required for the biochemical characterization of the membrane-bound enzyme, as it was difficult without due to the low native expression level and the enzyme instability in detergent solution.

### pBBR1MCS derivatives—the major plasmid family used in *Gluconobacter*

Besides testing the various plasmids described above for recombinant expression, in 2002, the plasmid pBBR122 was used to test whether lactose metabolism could be established in *G. oxydans* by cloning and expressing the *E. coli lacZY* genes from P_*lac*_ in five *G. oxydans* strains and one *Ga. liquefaciens* strain (Mostafa et al. 2002). In three *G. oxydans* strains the LacZ activities were similarly high as the LacZ activity in induced *E. coli* XL1-Blue cells, while on other *G. oxydans* strains and in *Ga. liquefaciens* the LacZ activities were much lower. Albeit for unknown reasons only a few transformants of *G. oxydans* were able to grow on lactose, these results demonstrated that active β-galactosidase can be produced in *G. oxydans* at levels much higher than the LacZ activities obtained with Tn*951* mentioned above. This also suggested that the Tn*951* promoter is very weak in *Gluconobacter* which could be useful when very low target gene expression levels are required.

The plasmid pBBR122 is based on the very small endogenous plasmid pBBR1 (2.6 kb) isolated from *Bordetella bronchiseptica* (Antoine and Locht 1992). pBBR122 was commercialized by MoBiTec and exhibits a broad-host-range compatible with IncP, IncQ, and IncW group plasmids as well as with ColE1- and p15A-based replicons. The pBBR1 derivative pBBR1MCS carrying pBBR1 *ori*, a MCS and a chloramphenicol resistance cassette was used to create four new derivatives each with a different antibiotic resistance cassette: pBBR1MCS-2 confers kanamycin resistance, pBBR1MCS-3 tetracycline resistance, pBBR1MCS-4 ampicillin resistance, and pBBR1MCS-5 gentamicin resistance (Kovach et al. 1995; Kovach et al. 1994). The family members exhibit several advantages in that they are relatively small (<5.3 kb), possess an extended MCS, allow direct selection of recombinant plasmids in *E. coli* via disruption of the LacZα peptide, and are mobilizable when the RK2 transfer functions are provided in *trans*, yet can also be introduced by electroporation.

Since 2006, the pBBR1MCS-based plasmids have been used in most *Gluconobacter* studies reporting recombinant gene expression; thus, it is the major expression plasmid lineage used in *Gluconobacter* and it also exhibits the most use cases in AAB in general (Table 1). While pBBR1MCS-3 providing tetracycline resistance was apparently not used in *Gluconobacter*, pBBR1MCS-5 has been used in 27 studies, pBBR1MCS-2 in 25 studies, and pBBR1MCS-4 in 8 studies. In 51 cases *Gluconobacter* genes, in 5 cases genes from other AAB genera, and in 13 cases genes from bacteria other than AAB, were expressed from P_*lac*_ or selected promoters (Table 2). In a few studies, fusion proteins with signal peptides for export or for surface display and a tagged protein for one-step membrane protein purification from *Gluconobacter* cells were produced.

**Table 1:** The five most frequently used plasmid lineages for recombinant target gene expression in AAB and the flexible SEVA plasmid toolkit

Backbone	Plasmid/Derivative	Reference	AAB genera use	#	AAB references
pBBR1	pBBR122, Cm^R^, Km^R^	Antoine and Locht 1992	*Komagataeibacter*	5	^a^ pBBR122 *K.* ref.
			*Gluconobacter*	1	Mostafa et al. 2002
			*Gluconacetobacter*	1	Mostafa et al. 2002
	pBBR1MCS, Cm^R^, P_*lac*_, P_T3_, P_T7_	Kovach et al. 1994	*Gluconacetobacter*	1	Rouws et al. 2010
	pBBR1MCS-5, Gm^R^, P_*lac*_, P_T3_, P_T7_	Kovach et al. 1995	*Gluconobacter*	27	Table 2
			*Gluconacetobacter*	1	Camelo et al. 2020
	pBBR1MCS-2, Km^R^, P_*lac*_, P_T3_, P_T7_	Kovach et al. 1995	*Gluconobacter*	25	Table 2
			*Acetobacter*	1	Gao et al. 2020a
	pBBR1MCS-4, Ap^R^, P_*lac*_, P_T3_, P_T7_	Kovach et al. 1995	*Gluconobacter*	8	Table 2
			*Acetobacter*	1	Wu et al. 2017
	pBBR1MCS-3, Tc^R^, P_*lac*_, P_T3_, P_T7_	Kovach et al. 1995	*Gluconacetobacter*	1	Velazquez-Hernandez et al. 2011
pUC18 & pMV329	pMV24, Ap^R^, P_*lac*_	Fukaya et al. 1989	*Acetobacter*	13	^b^ pMV24 *A*. ref.
			*Komagataeibacter*	4	^c^ pMV24 *K*. ref.
pUC18 & pAH4	pSA19, Ap^R^, P_*lac*_	Tonouchi et al. 1994	*Komagataeibacter*	8	^d^ pSA19 ref.
pTrc99A & pFF6	pTA99, Ap^R^, P_*trc*_ pTI99, Km^R^, P_*trc*_	Tajima et al. 2001 Hu et al. 2010	*Komagataeibacter*	6	^e^ pTA99 / pTI99 ref.
pUC19 & pDN19	pCM62, Tc^R^ , P_*lac*_	Marx and Lidstrom 2001	*Acetobacter*	6	^f^ pCM62 ref.
pSEVA	flexible design	Durante-Rodriguez et al. 2014	*Komagataeibacter*	7	^g^ pSEVA ref.

^a^pBBR122 *K.* ref.: Chien et al. 2006; Setyawati et al. 2007; Setyawati et al. 2009; Yadav et al. 2010; Liu et al. 2018

^b^pMV24 *A*. ref.: Fukaya et al. 1989; Fukaya et al. 1990; Fukaya et al. 1993; Takemura et al. 1993b; Takemura et al. 1993a; Kondo et al. 1995; Kondo and Horinouchi 1997; Kashima et al. 1999; Kashima et al. 2000; Okamoto-Kainuma et al. 2002; Okamoto-Kainuma et al. 2004; Nakano et al. 2004; Zheng et al. 2018

^c^pMV24 *K*. ref.: Iida et al. 2008a; Iida et al. 2008b; Iida et al. 2009; Konjanda et al. 2019

^d^pSA19 ref.: Tonouchi et al. 1995; Tonouchi et al. 1998b; Tonouchi et al. 1998a; Nakai et al. 1998; Nakai et al. 1999; Nakai et al. 2002; Ishida et al. 2002; Nakai et al. 2013

^e^pTA99 / pTI99 ref.: Fujiwara et al. 1992; Hu et al. 2010; Sunagawa et al. 2013; Fang et al. 2015; Jacek et al. 2018; Jacek et al. 2019

^f^pCM62 ref.: Deeraksa et al. 2006; Masud et al. 2010; Soemphol et al. 2011; Theeragool et al. 2018; Yakushi et al. 2018a; Phathanathavorn et al. 2019

^g^pSEVA ref.: Florea et al. 2016a; Florea et al. 2016b; Walker et al. 2019; Teh et al. 2019; Huang et al. 2020; Hur et al. 2020; Liu et al. 2020b

**Table 2:** Use of the pBBR1MCS-based plasmid family in *Gluconobacter*. The studies are grouped by the pBBR1MCS derivative and ordered by the year of publication. *cyt.* cytochrome; *DH* dehydrogenase; *DHA* dihydroxyacetone; *GFP* green fluorescent protein; *P* promoter; *PPP* pentose phosphate pathway; *PQQ* pyrroloquinoline quinone; *SNP* single nucleotide polymorphism; *SP* signal peptide

Expressed genes	Encoded activity/function	P ^a^	Context	Reference
pBBR1MCS-5				
*tldD*, *pqqA*, *pqqABCDE* from *G. oxydans* 621H	PQQ biosynthesis-related enzymes	P_*tufB*_,	analysis of PQQ biosynthesis genes in *G. oxydans*	Hölscher and Görisch 2006
		P_*pqqA*_,		
		P_*pqqBCDE*_		
*gdh, ga5dh* from *G. oxydans* 621H	membrane-bound PQQ-dependent glucose DH and gluconate-5-DH	P_*lac*_	increase of gluconate and 5-keto-d-gluconic acid production by *G. oxydans* strains	Merfort et al. 2006b
*ga5dh* from *G. oxydans* 621H	membrane-bound PQQ-dependent gluconate-5-DH	P_*tufB*_,	increase of 5-keto-d-gluconic acid production by *G. oxydans* mutant strain	Merfort et al. 2006a
		P_*gdh*_		
*sldAB* from *G. oxydans* 621H	membrane-bound PQQ-dependent polyol DH	P_*tufB*_,	increase of DHA production from glycerol by *G. oxydans* mutant	Gätgens et al. 2007
		P_*gdh*_		
*sldAB* from *G. oxydans* M5	membrane-bound PQQ-dependent d-sorbitol DH	P_*lac*_	characterization of the enzyme in *G. oxydans* M5 and role in miglitol precursor production	Yang et al. 2008
*sldBA* from *G. suboxydans* IFO 3255	membrane-bound PQQ-dependent d-sorbitol DH	P_*lac*_,	characterization of two membrane-bound d-sorbitol DHs and complementation in *G. frateurii*	Soemphol et al. 2008
		P_*sldBA*_		
*sldA’B’* from *G. oxydans* 621H	N-terminal fragments of membrane-bound PQQ-dependent polyol DH	P_*tufB*_	construction of the pEXGOX family; a cloning and expression vector system with P_*tufB*_ from *G. oxydans*	Schleyer et al. 2008
*adhAB* from *G. oxydans* DSM 2003	membrane-bound PQQ-dependent alcohol DH (mADH)	P_*lac*_	enzyme characterization; mADH mutant complementation; lactic acid production from 1,2-propanediol	Wei et al. 2010
*gdh* from G. oxydans M5	membrane-bound PQQ-dependent glycerol DH	P_*lac*_	enhanced production of DHA by *G. oxydans* mADH mutant	Li et al. 2010b
*vgb* from Vitreoscilla	*Vitreoscilla* hemoglobin	P_*tufB*_	improvement of cell growth and DHA production by *G. oxydans* M5	Li et al. 2010a
*adhAB* from *G. oxydans* DSM 2003	membrane-bound PQQ-dependent alcohol DH (mADH)	P_*adh*_	characterization of mADH and complementation in *G. oxydans* DSM 2003 mADH mutant	Wei et al. 2012
*xdh* from *G. oxydans* NH-10; *gdh* from *B. subtilis*	xylitol DH (XDH) and glucose DH (GDH)	P_*tufB*_	improvement of xylitol production by construction of a *xdh* and *gdh* co-expressing *G. oxydans* strain	Zhang et al. 2013
*ndh* and *ga2dh* from *G. oxydans* DSM 2003; *gfp*	membrane-bound type II NADH DH; FAD-dependent gluconate-2-DH; GFP	P_gHp0169_*,*	evaluation of promoter gHp0169 and improvement of 2-keto-d-gluconic acid production by *G. oxydans* DSM 2003	Shi et al. 2014
		P_*tufB*_		
*ga2dh* from *G. oxydans* DSM 2003	FAD-dependent gluconate-2-DH	P_*tufB*_	rational mutagenesis to increase the pBBR1MCS-5 copy number; 2-keto-d-gluconic acid production	Shi et al. 2015
*sldAB* from *G. oxydans* 621H; *gfp*	membrane-bound PQQ-dependent polyol DH; green fluorescent protein	P_GOX0169_, P_GOX0264_, P_GOX0452_, P_*tufB*_	promoter strength analysis and 5-keto-d-gluconate accumulation using combinatorial metabolic engineering strategies in industrial strain *G. oxydans* ZJU2	Yuan et al. 2016a
*ga2dh* from G. oxydans DSM 2003	FAD-dependent gluconate-2-DH	P_*tufB*_, P_*ga2dh*_, P_gHp0169_	improvement of 2-keto-d-gluconic acid production by *G. oxydans* DSM 2003	Li et al. 2016a
*adhS* and *adhABS* from *G. oxydans* DSM 2003	membrane-bound PQQ-dependent alcohol DH (mADH)	P_*adhS*_, P_*adhAB*_	study of the AdhS subunit and effect on mADH activity in *G. oxydans* DSM 2003; hydroxy acid production	Zhang et al. 2016
*gcd* from *X. campestris* DSM 3586	alcohol DH	P_GOX0169_	improvement of 5-keto-d-gluconate production by *G. oxydans* ZJU2 mutant strain	Yuan et al. 2016b
*zwf*, *gnd* from *G. oxydans* NH-10	glucose-6-phosphate DH and 6-phospho-gluconate DH	P_*tufB*_	improvement of xylitol production by *G. oxydans* NH-10; coenzyme regeneration efficiency of the PPP	Li et al. 2016b
fusion of L-RI gene from *Acinetobacter* sp. DL-28; *sdh* from *G. oxydans* DSM 2003; *gfp*	l-ribose isomerase (L-RI) C-terminally fused to PDZ ligand; membrane-bound PQQ-dependent d-sorbitol DH (SDH) N-terminally fused to PDZ domain; GFP fusion protein	P_*tufB*_	protein localization; novel strategy for the production of l-erythrose by localizing the assembly of L-RI to SDH *via* protein-peptide interaction of PDZ ligand and PDZ domain	Zou et al. 2017
*adh*, *idh*, *aldh*, *gidh*, *gdh*, *pqq3*, *pqq4*, *sldAB* from *G. oxydans* 621H	membrane-bound DHs (mDHs)	P_GOX1067-68_, P_GOX1857_,	test of promoter strength and enzymatic characterization of mDHs in *G. oxydans* multi-deletion strain BP.9	Mientus et al. 2017
genes of putative membrane-bound DHs from mother of vinegar DNA	glucose DH like DH; alcohol DH like DH; polyol DH like DH; aldehyde DH like DH; d-lactate DH like DH	P_*adh*_ (P_GOX1067-68_)	heterologous expression of metagenomic mDHs in *G. oxydans* BP.9 and identification of new substrate specificities	Peters et al. 2017
*adhA*, *adhAB*, *aldh* from *G. oxydans* DSM 2003	membrane-bound alcohol DH (mADH) and aldehyde DH (mALDH)	P_*tufB*_	characterization of mADH and mALDH; 3-hydroxypropionic acid production	Zhu et al. 2018
*sldAB* from *G. oxydans* ZJB-605	membrane-bound PQQ-dependent d-sorbitol DH	P_gHp0169_	improvement of miglitol precursor production by industrial strain *G. oxydans* ZJB-605	Ke et al. 2019
*trx* from *G. oxydans* NL71	thioredoxin	P_*lac*_	enhancement of *G. oxydans* resistance to lignocellulosic-derived inhibitors; xylonic acid production	Shen et al. 2020
*araC*, *araE* from *E. coli* MC4100	transcriptional regulator AraC; l-arabinose-transporter AraE	P_*araBAD*_	plasmid-based tunable l-arabinose-inducible expression system for *G. oxydans*	Fricke et al. 2020
*pqqABCDE* and *tldD* from *G. oxydans* H-8	PQQ biosynthesis-related enzymes	P_gHp0169_	increase PQQ biosynthesis in repeated batch biotransformation for miglitol precursor production by mutant strain *G. oxydans* H-8	Liu et al. 2020a
pBBR1MCS-2				
*gdh* from *G. oxydans* 621H	membrane-bound PQQ-dependent glucose DH	P_*lac*_	gluconate and 5-keto-d-gluconic acid accumulation by *G. oxydans*	Merfort et al. 2006b
GOX1857 from *G. oxydans* 621H	membrane-bound PQQ-dependent inositol DH	P_GOX1857_	substrate and co-factor analysis of GOX1857 in *G. oxydans* 621H	Hölscher et al. 2007
*uidA* from *E. coli* DH5α	β-d-glucuronidase UidA	P_*lac*_, P_GOX0254_, P_GOX0452_	characterization of constitutive *G. oxydans* promoter strength	Kallnik et al. 2010
*sldA* from *G. oxydans* DSM 7145	membrane-bound PQQ-dependent polyol / glycerol DH	P_*sldB*_	characterization of *sldA* disruptant mutant and substrate analysis of the enzyme	Voss et al. 2010
*mgdh* from *G. oxydans* 621H	membrane-bound PQQ-dependent glucose DH	P_*mgdh*_	evolution for enhanced growth and biotransformations by *G. oxydans* at low glucose concentration	Zhu et al. 2011
*edd*-*eda* and *gnd* from *G. oxydans* 621H	6-phosphogluconate dehydratase; 2-keto-3-deoxy-6-phospho-gluconate aldolase; 6-phosphogluconate DH	P_GOX0384_	analysis of cytoplasmic fructose catabolism in a Δ*gnd* mutant lacking the oxidative PPP and Δ*edd* Δ*eda* mutant lacking the Entner-Doudoroff pathway	Richhardt et al. 2012
*cyoBACD*, *cydAB* and *cydABCD* of *G. oxydans* 621H	quinol terminal oxidases cytochrome *bo*_3_ and cytochrome *bd*	P_GOX0384_	influence of terminal oxidases on growth and yield of *G. oxydans* 621H	Richhardt et al. 2013
GOX0265 of *G. oxydans* 621H	membrane-bound PQQ-dependent glucose DH (mGHD) with C-terminal Strep-tag	P_GOX0452_	gluconate production; influence of mGDH on O_2_ consumption rate; membrane protein purification by Strep-tactin affinity chromatography	Meyer et al. 2013
*phoA* and *treA* from *E. coli* DH5α	alkaline phosphatase and trehalase fused to selected SPs	P_GOX0264_	SP screening in *G. oxydans* and broadening the substrate range of *G. oxydans* for growth on trehalose	Kosciow et al. 2014
*sldhAB* from *G. oxydans* WSH-003	membrane-bound PQQ-dependent sorbitol DH (SLDH)	P_*tufB*_	enhancing production of l-sorbose from d-sorbitol; increase *sldhAB* mRNA level by artificial poly-A/T tail	Xu et al. 2014
*ndh* from *G. oxydans* DSM 3504	type II NADH DH (NDH-2)	P_*ndh*_	consequence of an additional NADH DH paralog on growth of *G. oxydans* 621H	Kostner et al. 2015
*sdhCDAB*, *sdhE A. pasteurianus; sdhE* from *Serratia sp.*	succinate DH (SDH) and assembly factor SdhE for attachment of FAD to SdhA	P_GOX0384_, P_*sdhE-Ace*_, P_*sdhE-Ser*_	SdhE-dependent formation of a functional *A. pasteurianus* SDH in *G. oxydans* 621H	Kiefler et al. 2015
GOX1801 from *G. oxydans* 621H	succinic semialdehyde reductase (SSR) fused C-terminally to Strep-tag	P_GOX0264_	characterization of GOX1801 enzyme activities; the first SSR from an aerobic bacterium with high catalytic efficiency	Meyer et al. 2015
*sldh* from *G. oxydans* WSH-003; *egfp*	d-sorbitol DH; enhanced GFP (eGFP),	P_B932_2000_, P_*tufB*_	identification of strong promoters; comparison of P_B932_2000_ with P_*tufB*_ form *E. coli* and from *G. oxydans*; enhancing production of l-sorbose	Hu et al. 2015
GOX1432 from *G. oxydans* 621H	cytoplasmic NADP^+^-dependent mannitol DH fused C-terminally to Strep-tag II	P_GOX0264_	role of mannitol DHs in osmoprotection of *G. oxydans* 621H	Zahid and Deppenmeier 2016
*xynA* from B. subtilis	endo-1,4-β-xylanase fused N-terminally to the PelB SP	P_GOX0264_	xylan utilization; degradation of renewable organic materials and incomplete oxidation for production of value-added products	Kosciow et al. 2016
*pqqA*, *pqqB*, *pqqC*, *pqqD*, *pqqE*, *tldD* from *G. oxydans* WSH-003	PQQ biosynthesis proteins	P_*pqqA*_, P_*tufB*_	analysis of PQQ levels in *G. oxydans* WSH-003 and related conversion of d-sorbitol to l-sorbose	Wang et al. 2016
*sdhCDAB* and *sdhE* from *A. pasteurianus*; *sucCD* from *Ga. diazotrophicus*	succinate DH (SDH) and assembly factor SdhE for attachment of FAD to SdhA; succinyl-CoA synthetase	P_GOX0264_, P_*sdhE*_	used for fragment construction for chromosomal insertion of genes to obtain *G. oxydans* 621H with completed TCA cycle for increased biomass yield from glucose	Kiefler et al. 2017
*fdhSCL* from *G. japonicus*;	membrane-bound fructose DH; extracellular sucrase SacC fused N-terminally to the PelB SP	P_GOX0264_	production of 5-ketofructose from fructose or sucrose using genetically modified *G. oxydans* strains	Siemen et al. 2018
*sacC* from *Z. mobilis* ZM4				
*fdhSCL* from *G. japonicus* NBRC3260	membrane-bound fructose DH (Fdh)	P_GOX0264_	production of 5-ketofructose; online monitoring of the respiration activity; pulsed and continuous fructose feed	Herweg et al. 2018
*phoA* from *E. coli*; *oprF* from *P. aeruginosa* PAO1	alkaline phosphatase PhoA; first 188 aa of outer membrane porin OprF fused C-terminally to Strep-tag; OprF-PhoA fusion protein	P_GOX0264_, P_GOX0452_	surface display of PhoA in *G. oxydans* using OprF for delivery and test of linker regions in OprF-PhoA fusion proteins	Blank and Schweiger 2018
codon-optimized *sldh* from *G. oxydans* G624; *lrenox* from *Lactobacillus reuteri*	sorbitol DH; NAD(P)H oxidase	P_*adh*_	overcoming inhibitory effect of NADPH formation during conversion of d-sorbitol to l-sorbose in *G. oxydans* KCTC 1091	Kim et al. 2019
*fdhSCL* from *G. japonicus* NBRC3260; *inv1417* from *G. japonicus* LMG 1417	membrane-bound fructose DH (Fdh); β-fructofuranosidase / invertase	P_GOX0264_	production of 5-ketofructose (5-KF) by *G. oxydans* and *G. japonicus*; production of 5-KF from sucrose by invertase and Fdh with recombinant *G. oxydans* strains	Hoffmann et al. 2020
*fdhSCL* from *G. japonicus* NBRC3260	membrane-bound fructose DH (Fdh)	P_GOX0264_	production of 5-ketofructose from sucrose by fructose DH and invertase with *G. oxydans* IK003.1	Battling et al. 2020
*levS1417* from *G. japonicus* LMG 1417	levansucrase	P_GOX0264_	high yield production of levan-type fructans by *G. japonicus* LMG 1417	Hövels et al. 2020
pBBR1MCS-4				
*sldAB* from *G. oxydans* 621H	membrane-bound sorbitol DH (mSLDH)	P_*tufB*_, P_*sldAB*_	growth performance and DHA production by engineered *G. oxydans* 621H	Lu et al. 2012
*fdhSCL* from *G. japonicus* NBRC3260	flavoprotein-cyt. c complex fructose DH (FDH)	P_*adhAB*_	production of FDH in *G. oxydans* NBRC12528 strains; 5-keto-d-fructose formation	Kawai et al. 2013
*aroQ* from *G. oxydans* NBRC3244	type II 3-dehydroquinate dehydratase	P_*lac*_, P_*aroQ*_	biotransformation of quinate to 3-dehydroshikimate by *G. oxydans* NBRC3244	Nishikura-Imamura et al. 2014
*kgdSLC* from *G. oxydans* NBRC3293	2-ketogluconate DH	P_*adhAB*_	production of 2,5-diketo-d-gluconate by *G. japonicus* NBRC3271	Kataoka et al. 2015
*sldBA* from *G. frateurii* NBRC101659	membrane-bound PQQ-dependent glycerol / polyol DH (GLDH)	P_*lac*_	sugar oxidase activities of GLDH in *G. frateurii* NBRC101659 strains	Yakushi et al. 2018c
*steP* from *G. frateurii* NBRC101659	sugar-transporting / exporting permease	none, P_*lac,*_ P_GOX0284_, P_GOX0452_	SNP in *steP* affects trehalose efflux / sorbose uptake; NADPH/NADP^+^ ratio and thermotolerance of *G. frateurii*	Matsumoto et al. 2018
*quiA* from *G. oxydans* NBRC3292 and NBRC3244	membrane-bound PQQ-dependent quinate DH (QDH)	P_lac_, P_*adhAB*_, P_*quiA*_	improving QDH activities, tested in *G. oxydans* NBRC12528 and *G. frateurii* NBRC101659 strains	Yakushi et al. 2018b
*sndh* from *Ga. liquefaciens* RCTMR10	membrane-bound sorbosone DH (SNDH)	P_*adhAB*_	expression in *G. oxydans* NBRC12528 and identification of the prosthetic group of SNDH	Yakushi et al. 2020

^a^If no promoter or no native upstream region of the cloned gene(s) is mentioned in the publication, promoter P_*lac*_ present on the pBBR1MSC-based plasmids was assumed to be responsible for target gene expression

### Other expression plasmids recently used in *Gluconobacter*

In parallel to the use of the very successful pBBR1MCS-based plasmid family in *Gluconobacter*, further plasmids have been constructed based on the two homologous cryptic plasmids pGOX3 (14.5 kb) found in two *G. oxydans* strains and pUC18 or pUC19 carrying pBR322 *ori*. The chimeric plasmids are selectable by conferring ampicillin resistance, are compatible with the pBBR1MCS family and double selectable except with pBBR1MCS-4 also carrying ampicillin resistance. In the first case, the *par* and *rep* loci of pGOX3 from *G. oxydans* DSM 2003 were amplified as a 2.3-kb fragment and cloned into pUC18, resulting in the 5 kb shuttle vector pZL1 (Zhang et al. 2010). Plasmid pZL1 was found to replicate in both *E. coli* and *G. oxydans* DSM 2003 and was maintained for 144 h during serial subcultures in the absence of selective pressure in 80% of the DSM 2003 cells, while pUC18 was almost completely lost already after 48 h. The relative plasmid copy number of pZL1 in DSM 2003 was found to be 13 times higher than that of the endogenous pGOX3. The capability of pZL1 to express heterologous genes in DSM 2003 was shown by using the fluorescence reporter gene *wasabi*. When expressed in DSM 2003 from the strong promoter P_*tufB*_, the fluorescence signal with the reporter plasmid pZL1-tufB-wasabi was almost as high as with pBBR1MCS-5-tufB-wasabi.

A comparable assembly process as for pZL1 was used to construct the shuttle vector pGUC based on the *par* and *rep* loci of pGOX3 from *G. oxydans* 621H and pUC18 (Gao et al. 2014). Plasmid pGUC and again the strong promoter P_*tufB*_ were used to express and test different combinations of five l-sorbose dehydrogenase (SDH) genes and two l-sorbosone dehydrogenase (SNDH) genes from *Ketogulonicigenium vulgare* in *G. oxydans* WSH-003, an industrial strain used for the conversion of d-sorbitol to l-sorbose. As the production of the vitamin C precursor 2-keto-l-gulonic acid from d-sorbitol involves three sequential oxidation reactions, the spatial proximity of the corresponding dehydrogenases appeared to be useful to enhance the production. Therefore, with a series of linker peptides, SDH-SNDH fusion enzymes were tested and for a selected SDH-SNDH fusion the *pqqA* gene alone or the *pqqABCDE* gene cluster for PQQ biosynthesis was additionally included on the pGUC plasmid. Additional expression of the PQQ biosynthesis gene(s) enhanced cell growth and with the stepwise metabolic engineering of *G. oxydans*, the final 2-keto-l-gulonic acid titer was improved 8-fold (39 g/L) compared to that obtained with the independent expression of the dehydrogenase genes (Gao et al. 2014). In further work, the adaptor protein SH3 gene sequence was fused via a linker sequence to the SDH gene of the SDH-linker-SNDH construct and co-expressed with the gene of the small trimeric protein CutA from *Pyrococcus horikoshii* fused to the SH3lig gene sequence, all present on pGUC and each expressed from P_*tufB*_ (Gao et al. 2020c). The adaptor protein SH3 as docking protein and its ligand SH3lig as docking station peptide improved the chemical structure stability of fused SDH-SNDH complexes surrounding the trimeric CutA protein. The recombinant strain *G. oxydans* WSH-003 with the pGUC-based expression plasmid produced 40 g/L of 2-keto-l-gulonic acid after 168 h. Additionally, co-expression of the *pqqABCDE* operon from P_*tufB*_ on pGUC increased the 2-keto-l-gulonic acid titer to 43 g/L, demonstrating an efficient conversion of d-sorbitol to 2-keto-l-gulonic acid with a single strain (Gao et al. 2020c).

In another study, the amplified *par*-*rep* fragment of pGOX3 from *G. oxydans* 621H was ligated with pUC19 and shuttle vector pUCpr was obtained (Yuan et al. 2016a). In this work two compatible plasmids, pBBR1MCS-5 and pUCpr have been applied simultaneously in the same strain for target gene expression. Plasmid pBBR1MCS-5 was used to express the membrane-bound PQQ-dependent sorbitol dehydrogenase genes *sldAB* from the strong promoter P_GOX0169_ in different combination with pUCpr derivatives carrying either the PQQ biosynthesis genes *pqqABCDE* or *pqqABCDE-tldD* under control of P_GOX0169_ alone, or each in combination with the cytochrome *bo*_3_ oxidase genes *cyoBACD* expressed from a separate copy of the P_GOX0169_ promoter. In fed-batch cultivation with pH and dissolved oxygen tension control, 5-keto-d-gluconate production could be significantly enhanced with the combinatorial metabolic engineering strategy to 162 g/L based on the two-plasmid-system in the recombinant industrial *G. oxydans* strain ZJU2 (Yuan et al. 2016a).

### Plasmid-based promoter analysis in *Gluconobacter*

Besides the expression of target genes in screening, complementation, functional, or metabolic engineering studies, plasmids have also been used in *Gluconobacter* to analyze and compare relative promoter strength using selected reporter genes. The promoter probe vector pCM130 is a derivative of the P_*lac*_ expression plasmid pCM62 introduced below in the *Acetobacter* section and carries the reporter gene *xylE* encoding catechol 2,3-dioxygenase from *Pseudomonas* in place of P_*lac*_. It was used to compare the strength of the promoter P_*pqqA*_ and a potential intrinsic promoter P_*pqqBCDE*_ of the *pqqABCDE* operon from *G. oxydans* 621H (Hölscher and Görisch 2006). With both d-mannitol or d-gluconate as a growth substrate, the reporter activities of P_*pqqBCDE*_ were below the activity found for the pCM130 empty vector, while with P_*pqqA*_ the reporter activities were at least 3.5-fold higher than with the empty vector. Therefore, the reporter assay results suggested that P_*pqqA*_ represents the only promoter of the *pqqABCDE* operon in *G. oxydans* 621H, a situation similar to that found for other PQQ-synthesizing bacteria.

With the aim to provide effective P_*lac*_-independent expression vectors for gene expression in *G. oxydans*, constitutive promoters were selected to determine their relative strength using pBBR1MCS-2 and the β-d-glucuronidase gene *uidA* from *E. coli* as a reporter (Kallnik et al. 2010). The promoters of GOX0264 and GOX0452 encoding the ribosomal proteins L35 and L13, respectively, were found to be strong (P_GOX0264_) and moderate (P_GOX0452_), while the intrinsic promoter P_*lac*_ of pBBR1MCS-2 appeared rather weak in *G. oxydans* 621H. In another approach to identify strong promoters, chromosomal DNA of *G. oxydans* DSM 2003 was randomly digested and DNA fragments were inserted into pBBR1MCS-5-gfp carrying the promoterless green fluorescent protein gene as a reporter (Shi et al. 2014). After screening the GFP fluorescence signals of 710 transformants, the one with the highest fluorescence intensity was selected and a 261-bp DNA fragment was obtained that exhibited promoter activity and was homologous to the *G. oxydans* 621H intergenic region between GOX0168 encoding an NAD-dependent DNA ligase and GOX0169 encoding a hypothetical protein. The promoter region was termed gHp0169 and P_gHp0169_ activity was determined to be almost 2-fold stronger (2.47 U/mg) than P_*tufB*_ from *G. oxydans* (1.39 U/mg) when assayed with pBBR1MCS-5 and the *ndh* gene encoding the type II NADH dehydrogenase as a reporter.

Since strong promoters are one of the essential factors that can improve strain performance by overexpression of specific genes, a pipeline for screening strong promoters by proteomics analysis and promoter assays was also established (Hu et al. 2015). Using this approach, the strong promoter P_B932_2000_ was identified in *G. oxydans* WSH-003. As assessed by analysis of pBBR1MCS-2-based *egfp* expression and qRT-PCR analysis of *egfp* mRNA, the strength of P_B932_2000_ in *G. oxydans* WSH-003 was at least 2-fold higher than that of P_*tufB*_ from *G. oxydans* and at least 4-fold higher than that of P_*tufB*_ from *E. coli*. In a further study the aforementioned *G. oxydans* promoters P_*tufB*_, P_GOX0169_, P_GOX0264_, and P_GOX0452_ were assayed for their relative strength in *G. oxydans* 621H using pBBR1MCS-5 and *gfp* as reporter gene (Yuan et al. 2016a). In this assay, P_GOX0169_ was found to be the strongest one, followed by P_GOX0264_, P_*tufB*_, and P_GOX0452_, respectively.

In contrast to the previous studies searching mainly for strongest promoters, another study aimed to develop a new vector for successful expression of genes encoding membrane-bound dehydrogenases in *G. oxydans*, which typically requires intermediate or even low expression levels to obtain functional enzymes (Mientus et al. 2017). In this study, the strength and the regulation of the promoters of the alcohol dehydrogenase gene (P_*adh*_) and the inositol dehydrogenase gene (P_*idh*_) were analyzed using pBBR1MCS-5-based pJV17 derivatives carrying the *E. coli lacZ* reporter gene. According to the β-galactosidase assays, both promoters were practically not active in *E. coli*, while they showed good activity in *G. oxydans* grown on d-mannitol, d-sorbitol or d-glucose as carbon source. P_*adh*_ led to similar LacZ activities with all three substrates and was generally more active than P_*idh*_, which led to weak LacZ activity on d-mannitol and appeared to be repressed 100-fold in the presence of d-glucose when compared to d-sorbitol.

### Chromosomal integrations and intergenic regions suitable for target gene expression

Besides plasmid-based expression, chromosomally integrated expression cassettes using either the native promoter or selected endogenous or heterologous promoters have also been applied for target gene expression in *Gluconobacter*. In an early study analyzing the maltose-oxidizing ability of a *G. oxydans* strain, the effect of a single amino acid substitution on the substrate specificity of the PQQ-dependent glucose dehydrogenase (mGDH) was tested by chromosomal insertion due to the lack of a suitable expression plasmid for *Gluconobacter* at that time (Cleton-Jansen et al. 1991). A *G. oxydans* strain with an endogenous mGDH not capable of oxidizing maltose was transformed with the non-replicable pGP173 plasmid backbone, which carried the mGDH gene of the maltose-oxidizing strain under control of its native promoter region and a kanamycin resistance cassette. Recombinant cells formed by homologous recombination were selected by growth on maltose. Further analysis revealed that the exchange H787N broadened the substrate spectrum of the mGDH and enabled the oxidation of the disaccharide maltose.

Later, chromosomal integrations for target gene expression have been used in combinatorial metabolic engineering studies for which suitable integration sites had to be selected. For example, to increase the biomass yield of *G. oxydans* 621H on glucose, of which 90% is typically oxidized already in the periplasm to gluconate and 2-ketogluconate accumulating unused in the medium, the carbon flux into the central carbon metabolism needs to be increased and additionally, the incomplete tricarboxylic acid (TCA) cycle of *G. oxydans* 621H needs to be completed by a succinate dehydrogenase and a succinyl-CoA synthetase, since these two TCA cycle enzymes are absent. These requirements were addressed by combining the chromosomal insertion of several heterologous genes and deletion of the two glucose dehydrogenase genes to eliminate the periplasmic and cytosolic oxidation of glucose to gluconate (Kiefler et al. 2017). In detail, the cytoplasmic NADP-dependent glucose dehydrogenase gene *gdhS* was replaced by the succinate dehydrogenase genes *sdhCDAB* and the flavinylation factor gene *sdhE* from *A. pasteurianus* with the *sdhCDAB* operon under the control of strong P_GOX0264_ and *sdhE* under the control of its native promoter. The membrane-bound PQQ-dependent glucose dehydrogenase gene *gdhM* was replaced by the succinyl-CoA synthetase genes *sucCD* from *Ga. diazotrophicus* again using the strong promoter P_GOX0264_. Furthermore, the pyruvate decarboxylase gene *pdc* was replaced by a second *ndh* gene for a type II NADH dehydrogenase with its native promoter from *G. oxydans* DSM 3504 to eliminate acetate formation from pyruvate and to increase the overall NADH oxidation capacity. Together, the biomass yield of the engineered plasmid-free *G. oxydans* strain IK003.1 was increased by 60% and the strain was stable for more than 70 generations, making it very interesting as a host for oxidative biotransformations and further metabolic engineering approaches (Kiefler et al. 2017; Kranz et al. 2017).

Generally, for successful expression of chromosomally integrated recombinant genes, suitable intergenic regions need to be identified. Consequently, *G. oxydans* IK003.1 was used to test different intergenic regions (IGRs) selected on the basis of RNAseq data for their suitability to integrate and express the fructose dehydrogenase (FDH) genes *fdhSCL* from *G. japonicus* under the control of the strong promoter P_GOX0264_ for the oxidative biotransformation of fructose to 5-ketofructose (5-KF) (Battling et al. 2020; Kranz et al. 2018). 5-KF is a promising non-nutritive natural sweetener for which high-titer production was achieved already before in plasmid-based approaches (Kawai et al. 2013; Siemen et al. 2018). In the chromosome-based approach, four IGRs have been identified in the *G. oxydans* 621H genome suitable for expression of heterologous genes. That is the IGR of the genes GOX0013-GOX0014, GOX0028-GOX0089; GOX0038-GOX0039 and GOX2095-GOX2096, respectively, all located close to the genomic *ori* to take advantage of the increased average DNA copy number during chromosome replication in the exponential growth phase. Finding more than one suitable integration site allowed independent double-integration of the *fdhSCL* operon associated with a strong increase in the 5-KF production rate compared to the single integration strains. Therefore, more integrated copies of the *fdhSCL* operon are expected to further increase the 5-KF production rate by plasmid-free strains. Additionally, the use of a promoter stronger than P_GOX0264_ could enhance *fdhSCL* expression and thereby 5-KF production. Besides fructose as substrate, 5-KF could also be produced from the cost-efficient and renewable feedstock sucrose when applying a sucrose-hydrolyzing enzyme and FDH (Hoffmann et al. 2020). The *fdhSCL* genes were disruptively integrated into the mGDH gene locus under the control of the strong promoter P_GOX0264_ and the invertase Inv1417 gene was expressed under the control of the same promoter on a pBBR1MCS-2-based plasmid. The average yield of 5-KF formation from sucrose was 90 mM 5-KF, which is 84% in relation to the fructose released from 105 mM sucrose almost completely hydrolyzed. The invertase contains a Tat-signal peptide for secretion into the periplasm, but only 40% of the total invertase activity was found in the periplasm and 60% in the cytoplasm. This suggested that plasmid-based expression of the Inv1417 gene using the strong promoter P_GOX0264_ possibly caused an overload of the Tat export machinery. Chromosomal integration of the invertase gene into one of the suitable *G. oxydans* IGRs mentioned above might solve this limitation.

The same principle of combining gene deletion to eliminate undesired enzyme activity and inserting an expression cassette into the disrupted gene locus was applied for biotransformation of glucose to 2-keto-d-gluconic acid via intermediate d-gluconic acid by recombinant *G. japonicus*. The gene for a subunit of gluconate-5-dehydrogenase producing the unwanted by-product 5-keto-d-gluconic acid from d-gluconic acid was deleted and in this locus a cassette for the expression of a gluconate-2-dehydrogenase gene was inserted to increase 2-keto-d-gluconic acid formation from d-gluconic acid produced from glucose (Zeng et al. 2019).

### l-Arabinose-induced AraC-dependent regulatable expression

**Fig. 2:**
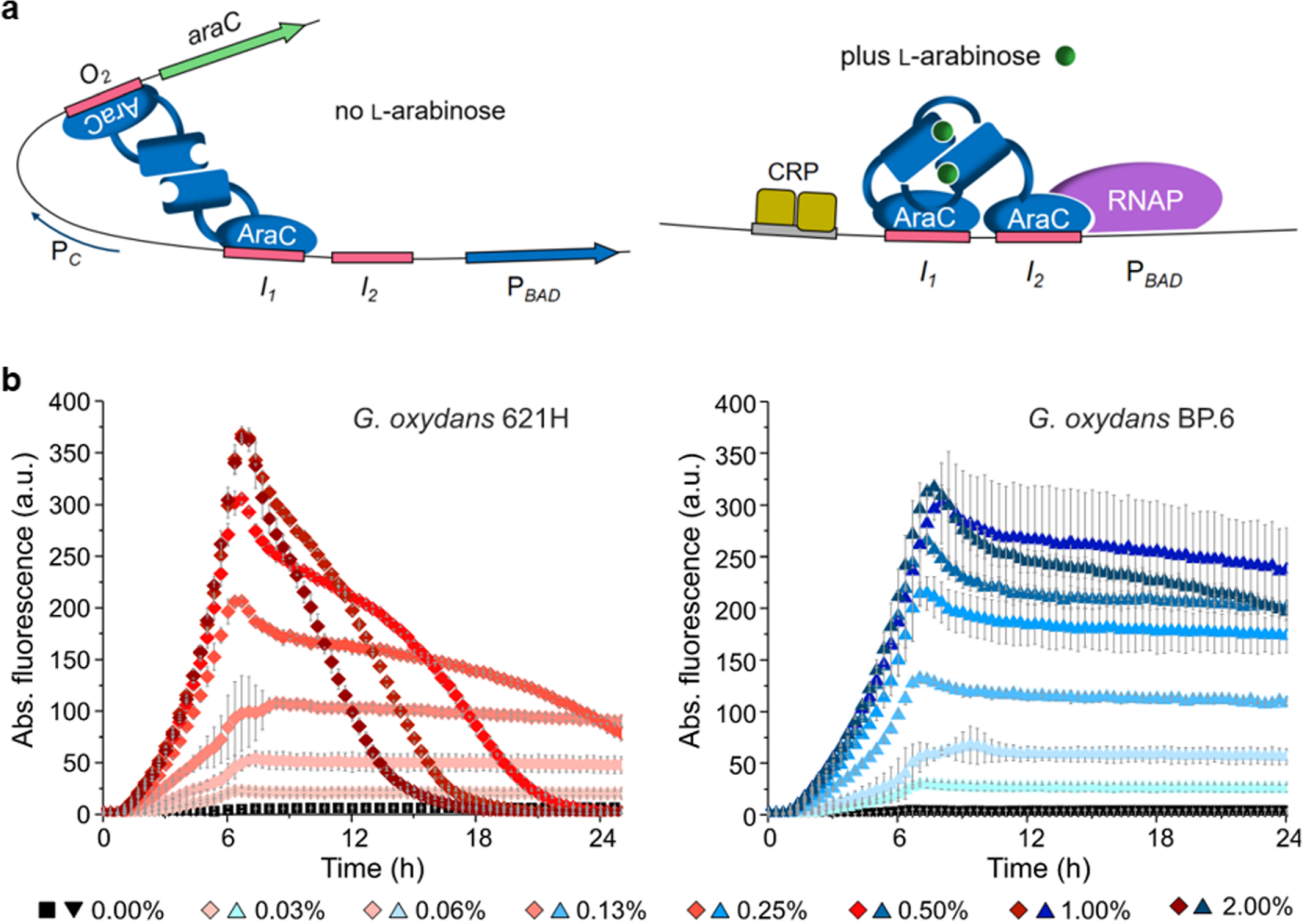
**a** The region of the P_*araBAD*_ promoter between the *araC* and *araBAD* genes illustrating the regulatory mode of action by the AraC regulator protein according to Schleif 2010. **b** AraC-dependent induction of mNeonGreen (mNG) expression up to 480-fold in *G. oxydans* 621H and mutant strain BP.6 both with plasmid pBBR1MCS-5-*araC-*P_*BAD*_*-mNG* induced by concentrations of l-arabinose (Fricke et al. 2020). As further analyzed and discussed in the study, the decrease of the mNG fluorescence in strain 621H at stronger induction is pH-dependent due to enzymatic oxidation of l-arabinose forming l-arabinonic acid and can be avoided by either using the multi-deletion strain BP.6 or pH-controlled condition

Until now, an l-arabinose-inducible *araC*-P_*araBAD*_ system on the pBBR1MCS-5 backbone is the only regulatable system available for *Gluconobacter* (Fricke et al. 2020). The mechanism of transcriptional gene regulation by AraC is different compared to those of the pure repressor proteins TetR and LacI, which just dissociate from their operator DNA when their respective inducer is bound to the protein. In *E. coli* AraC does not only repress P_*araBAD*_ by looping the DNA when bound to specific target sequences in the absence of l-arabinose, but also is essential for activation of P_*araBAD*_ (Fig. 2a). In the presence of l-arabinose, a modified binding of AraC to target sequences causes unlooping of the promoter DNA and stimulation of both the binding of RNA polymerase and the transition from the closed to the open promoter complex (Schleif 2010; Soisson et al. 1997). Additionally, in *E. coli*, the cAMP receptor protein (CRP) also plays an important role. According to the *G. oxydans* 621H genome sequences, a CRP appears to be absent (Kranz et al. 2017; Prust et al. 2005). The heterologous *araC*-P_*araBAD*_ system of *E. coli* MC4100 is very tight in *G. oxydans* even in the absence of AraC, indicating that promoter P_*araBAD*_ is not active in *G. oxydans* without AraC. The system is also well-tunable with up to 480-fold induction by l-arabinose concentrations up to 1% (w/v) (Fig. 2b). Unexpectedly, the performance of this system was strongly affected by only one or two small sequence alterations in the constructed plasmids. A single G to C exchange between the RBS and the ATG start codon of the reporter gene to create a *Nde*I cloning site in the MCS region of the *araC*-P_*araBAD*_ empty vector and/or the short remaining sequence of the *Xho*I site (CTCGAG) after the stop codon of the reporter gene, strongly reduced the maximum of the resulting reporter activity. In future studies, systematic analysis of such and other sequence alterations including terminators and orientations of neighboring genes (for example, target gene and the plasmid resistance cassette) may reveal specific rules that should be considered for successful construction and establishment of tight and regulatable heterologous expression systems in *Gluconobacter* or in AAB.

The l-arabinose concentrations required for highest induction in *G. oxydans* were much higher than the ones typically used for *E. coli*. Attempts to increase the arabinose sensitivity of the *araC*-P_*araBAD*_ system by expression of the l-arabinose transporter gene *araE* in tandem with *araC* from the promoter of *araC* had a strong negative effect on the growth of *G. oxydans* and the expression performance (Fricke et al. 2020). With a pBBR1MCS-5 derivative carrying *araE* separately under control of the weak constitutive *G. oxydans* promoter P_GOX0384_ no *G. oxydans* 621H transformants could be obtained yet, suggesting a severe growth defect upon *araE* expression in *G. oxydans*, even in the absence of l-arabinose (our unpublished results). Thus, before using AraE to hopefully increase the sensitivity of the *araC*-P_*araBAD*_ system toward l-arabinose, the strong negative effect of *araE* expression should be understood and overcome. Therefore, conditional and tuned expression of *araE* using the *araC*-P_*araBAD*_ system itself could help to clarify the negative effects of *araE* expression in *G. oxydans*. Likewise, *araC*-P_*araBAD*_-dependent conditional expression could also help to express genes for other “difficult” transporters and (membrane-bound) enzymes in AAB.

## Target gene expression in *Komagataeibacter*

Taken into account the changes in the AAB genera systematics and the associated transfers of species as described before, we found 42 studies for *Komagataeibacter* reporting on expression plasmids, among which 5 major lineages can be identified. Almost all studies employed electroporation as the method of choice for transfer of plasmids into *Komagataeibacter*, whereas conjugation was rarely used (Hall et al. 1992; Zhu et al. 2012).

### Early plasmid studies in *Komagataeibacter*

Initial work on the genetics to provide a gene transfer system was published by Johs Kjosbakken and co-workers in 1986 when studying the conjugative transfer of broad-host-range plasmids and cloning vectors (RP1, pKT210, pRK290, pBR322) with several antibiotic resistances into bacterial cellulose-producing *K. xylinus* (alias *Ga. xylinus* alias *A. xylinum*) and analyzing the number of transconjugants (Valla et al. 1986; Valla et al. 1987). The plasmid RP4::Mu *c*ts61 was used for the insertion of Tn*1* into the endogenous and conjugatively mobilizable 16-, 44-, and 64-kb plasmids of *K. xylinus*. Many of the Tn*1* insertions affected the copy number of the plasmids, yet it provided a selectable marker for the potential use of the plasmids in gene transfer experiments. In follow-up studies on cellulose-forming (Cel^+^) and cellulose-negative (Cel^-^) mutants of *K. xylinus* the broad-host-range cloning vector pVK100 and cosmid vector pRK311 were used to prepare gene libraries of *K. xylinus* to search for genes expressed from their native promoters and complementing the Cel^-^ phenotype (Fjaervik et al. 1991; Valla et al. 1989). The screenings revealed a uridine 5’-diphosphoglucose (UDPG) pyrophosphorylase gene designated *celA* involved in cellulose biosynthesis for which UDPG is the precursor, and a gene encoding phosphoglucomutase activity, which catalyzes the second step of UDPG synthesis from glucose by isomerization of glucose-6-phosphate to glucose-1-phosphate.

In a similar complementation screening, a cosmid vector derived from the high copy number, double-replicon vector pKT230, exhibiting a broad host range and found to be stably maintained in *K. xylinus*, was used for the construction of a genomic library (Wong et al. 1990). Four genes designated *bcsA*, *bcsB*, *bcsC*, and *bcsD*, which form the cellulose synthase operon, were found to complement the Cel^-^ phenotype caused by a cellulose synthase deficiency. In this study, it was also discovered that *K. xylinus* could be transformed by DNA via electroporation and a small endogenous cryptic plasmid (3.6 kb) was found. The latter was used to construct two shuttle plasmids by ligating the small cryptic plasmid with the *E. coli* plasmids pUC18 and pUC19 at the *Sst*I sites to obtain pUC18-824 and pUC19-824. These shuttle plasmids confer ampicillin resistance and also serve as expression vectors due to the presence of P_*lac*_ from *E. coli* which is actively transcribed in *K. xylinus*. Cloning of the single *bcs* genes expressed from P_*lac*_ allowed specific complementation of different Cel^-^ mutants. Additionally, replacing the native *bcs* promoter P_*bcs*_ on the chromosome with the heterologous promoters P_*lac*_ or P_*tac*_ reduced the cellulose synthase activities of cells to -60% and -85% of the level of the parental strain, demonstrating that the promoter P_*bcs*_ is quite strong and useful for high expression of desired target genes. In a later study on the cellulose-synthesizing operon *acs* in another *K. xylinus* strain, the cosmid vector pRK311 already mentioned above was used to express the single gene *acsD* from P_*tac*_ for recovery of normal cellulose production in an *acsD* mutant (Saxena et al. 1994).

### pUF106, pTA99, and pTI99—a first major expression plasmid lineage

The principle of fusing endogenous cryptic plasmids derived from *K. xylinus* strains with well-characterized *E. coli* plasmids to obtain shuttle vectors was also tested and applied in further studies using pUC18, pBR322 or the pBR322-based plasmid pTrc99A. Based on the endogenous *K. xylinus* plasmid pFF6 (2.72 kb) fused with pUC18 or with pTrc99A, the plasmids pUF106 and pTA99 were constructed to obtain shuttle vectors (Fujiwara et al. 1992; Tajima et al. 2001). Plasmid pTA99 was used to express the (exo)polysaccharide metabolism-related β-glucosidase gene *bglxA* from a *K. xylinus* strain driven by the strong *E. coli* P_*lac*_-P_*trp*_ hybrid promoter P_*trc*_. Analysis of culture supernatants demonstrated an approximately tenfold higher specific β-glucosidase activity compared to *K. xylinus* cells carrying the empty vector as a control. Similar to pTA99, shuttle vector pTI99 was also obtained by fusion of pFF6 with pTrc99A (Hu et al. 2010). It was used to clone various gene variants encoding N-terminally truncated AxCeSD proteins that form subunit D of the cellulose-synthesizing terminal complex in *K. xylinus* (Hu et al. 2010). Thereby, the N-terminal loop of subunit D, especially residue Lys6, turned out to be important for cellulose production. The AxCeSD and CcpAx proteins of the cellulose-synthesizing terminal complex were also expressed as fusion proteins with an enhanced green fluorescent protein (EGFP) using pTI99 (Sunagawa et al. 2013). In fluorescence microscopy analysis, the AxCeSD-EGFP fusion protein showed a cellular localization similar to the CcpAx fusion protein. Together with other data, AxCeSD and CcpAx showed significant interaction and were suggested to function as members of the terminal complex in *K. xylinus*.

To obtain modified bacterial cellulose with altered mechanical strength, biodegradability, and bioactivity for biomedical use, the curdlan synthase gene *crdS* from *Agrobacterium* was expressed in *K. xylinus* using pTI99 (Fang et al. 2015). It enabled curdlan (β-1,3-glucan) to be synthesized in *K. xylinus* simultaneously with cellulose nanofibers in vivo for biopreparation of nanocomposites. Production of bacterial cellulose with altered and advantageous properties could also be obtained without chemical modifications solely through altering the tight spatial organization of the cellulose fibers using a non-motile cellulose-producing *K. hansenii* with increased cell length and the ability to move on the surface of the medium (Jacek et al. 2019; Jacek et al. 2018). Therefore, the *motAB* genes encoding motor and stator proteins essential for flagellum rotation in numerous bacterial species were expressed either as an operon, or alone, or each gene as a translational fusion with *gfp* using the pTI99 vector backbone. It was assumed that probably the torque produced by the MotAB proton pump could affect other yet unknown motility mechanisms, cell division, filamentation or transport, thereby affecting the structure of the cellulose. Indeed, *K. hansenii* mutants with increased cell length and motility were shown to produce altered cellulose membranes with increased pore sizes and a relaxed fiber structure, which supported chondritic cell proliferation important for potential future application in tissue engineering.

### pSA19—a second major expression plasmid development

In parallel to the pFF6-based plasmid lineage, another endogenous cryptic plasmid termed pAH4 (4 kb) from a cellulose-producing *K. xylinus* strain was used for construction of a shuttle vector and established pSA19 as another major expression plasmid. *Hind*III-linearized pAH4 and pUC18 were fused to obtain the shuttle vector pSA19, which contains several pUC18 cloning sites and the promoter P_*lac*_ for expression of cloned genes (Tonouchi et al. 1994). The copy number of pSA19 in *K. xylinus* is roughly ten per genome and transformation efficiency was strongly increased when recombinant pSA19 plasmids were propagated in *K. xylinus* instead of *E. coli*, suggesting the presence of an effective restriction-modification system in *K. xylinus*. Later, restriction data obtained from two cryptic plasmids discovered in *K. xylinus* B42 showed that these plasmids contain protected *Eco*RI and *Apo*I sites. The protection was also present in the chromosomal DNA and the results suggest the presence of a modification system in *K. xylinus* that recognizes the tetranucleotide 5’-AATT (Petroni et al. 1996). However, plasmid pSA19 was successfully used in 8 further studies on cellulose-producing *K. xylinus* strains:

(i) pSA19 was used to express a wild-type endo-β-1,4-glucanase from *Bacillus* and a mutated variant (H131F), which is inactive but still binds to cellulose, to study their effects on cellulose production by *K. xylinus* (Tonouchi et al. 1995). The native glucanase enzyme enhanced cellulose production and reduced the amount of a polysaccharide called acetan, while the inactive variant did not affect cellulose production. It was concluded, therefore, that the endoglucanase activity itself, but not the cellulose-binding ability, was essential for the enhancement of cellulose production.
(ii) The extracellular sucrase gene *sucZE3* from *Zymomonas mobilis* together with the secretion-activating factor gene *zliS* and the *B. subtilis* levansucrase gene *sacB* containing a mutation causing decreased levan-forming activity were cloned into pSA19 under the control of P_*lac*_ to study cellulose production by *Komagataeibacter* from sucrose, which is the most suitable carbon source for the economical production of bacterial cellulose (Tonouchi et al. 1998b). The gene expression resulted in increased cellulose production and reduced levan accumulation.
(iii) A sucrose phosphorylase (SPase) gene from *Leuconostoc mesenteroides* was expressed with pSA19 using several promoters (P_*lac*_, P_*tac*_, P_*ugp*_ from *K. xylinus*, P_*gdh*_ from *G. oxydans*) to improve cellulose production in *K. xylinus* (Tonouchi et al. 1998a). Compared to expression from P_*lac*_, the SPase expression level was 78% with P_*tac*_, 37% with P_*ugp*_, and only 13% with P_*gdh*_. Interestingly, a small increase of the 5’-UTR length with a modified P_*lac*_ region increased the SPase expression level 3-fold.
(iv) pSA19 was used to express a sucrose synthase gene with the 5’-upstream region (~3.1 kb) of the cellulose synthase operon *bcs* from a *K. xylinus* strain instead of P_*lac*_ to study this *bcs* promoter region (Nakai et al. 1998). In *K. xylinus*, the expression occurred more than 241 bp upstream from the ATG start codon within the 1.1 kb upstream region. In *A. aceti* the expression was 75% of that in *K. xylinus*, while in *E. coli* no expression at all was detected, suggesting that the *bcs* upstream region studied may function as an ABB-specific promoter.
(v) To make use of the free energy of the glycosidic bond in sucrose for cellulose biosynthesis, the gene encoding native sucrose synthase from mung bean or a variant with a S11E mutation mimicking phosphorylation were cloned and expressed with pSA19 in *K. xylinus* under control of P_*lac*_ (Nakai et al. 1999). Sucrose synthase reversibly converts sucrose and UDP to fructose and UDP-glucose, the substrate of cellulose synthase. Expression of sucrose synthase in *K. xylinus* enhanced cellulose production from sucrose and the S11E variant with an increased sucrose affinity had an even stronger stimulating effect on cellulose synthesis.
(vi) For complementation of an ORF2 disruptant mutant of *K. xylinus* and functional analysis of the ORF2 polypeptide involved in cellulose synthesis, the ORF2 sequence plus a kanamycin resistance cassette was cloned into pSA19 (Nakai et al. 2002). The parental strain produced tough, colorless, and insoluble cellulose pellicles, whereas the ORF2 mutant produced thin, yellow, and fragile pellicles. The ORF2 polypeptide was suggested to be involved in the assembly of glucan chains into crystalline cellulose I microfibrils.
(vii) For complementation of *K. xylinus* mutants with disrupted *aceR* and *aceQ*, these genes involved in acetan biosynthesis were cloned into pSA19 for expression (Ishida et al. 2002). NMR and ESI-MS analyses of the produced water-soluble polysaccharides suggested that *aceQ* and *aceR* encode a glucosyltransferase and a rhamnosyltransferase, respectively.
(viii) In a mutant of *K. xylinus* in which the carboxymethylcellulase gene *cmcax* was disrupted by an ampicillin resistance cassette, the *cmcax* gene was expressed in *trans* using the pSA19 backbone carrying a kanamycin resistance cassette as an additional selection marker (Nakai et al. 2013).

### pBBR122 and pMV24—two other major expression plasmids used in *Komagataeibacter*

Another expression plasmid stably used in *Komagataeibacter* is the broad-host-range plasmid pBBR122 introduced above in the *Gluconobacter* section. With this plasmid, the gene of the bacterial hemoglobin from *Vitreoscilla* (VHb) was expressed driven by the constitutive *bla* promoter in cellulose-producing *K. xylinus* (Chien et al. 2006; Liu et al. 2018; Setyawati et al. 2007). VHb has been widely applied to improve cell survival during hypoxia. The hemoglobin was biochemically active also in *K. xylinus* and enhanced both the growth rate in shaken cultures by 50% and the cellulose titers. VHb allowed growth or survival of *K. xylinus* at lower oxygen tension and facilitated cellulose production in static culture, in which the polysaccharide exhibited interesting altered material properties. Cellulose nanofibers can also be used to self-immobilize *K. xylinus* in a biofilm, with the advantage of better resistance toward harsh biotransformation reaction conditions. This was tested for the production of α-ketoacids by a heterologous d-amino acid oxidase (DAAO), the gene of which was constitutively expressed from P_*lac*_ using pBBR122 (Setyawati et al. 2009). In the DAAO-catalyzed reaction toxic H_2_O_2_ is formed as a product. With self-immobilized *K. xylinus* cells expressing the DAAO gene, the system exhibited improved thermal and operational stability, as well as easy retrieval for repeated use. In contrast, for biomedical and biomass conversion applications, degradability of bacterial cellulose is important. To improve the poor in vitro and in vivo degradability of bacterial cellulose, a three-gene operon from *Candida albicans* for UDP-*N*-acetylglucosamine (UDP-GlcNAc) synthesis was expressed from the constitutive promoter P_*bla*_ in *K. xylinus* with the aim to introduce GlcNAc residues by cellulose synthase and produce a chimeric polymer (Yadav et al. 2010). The modified bacterial cellulose exhibited a high GlcNAc content and lower crystallinity, making it a multifunctional bioengineered polymer susceptible to lysozyme, an enzyme widespread in the human body, and to rapid hydrolysis by cellulase, an enzyme commonly used in biomass conversion.

Another shuttle vector used in *Komagataeibacter* studies from 2008 onward is pMV24 already constructed and introduced in 1989 for expression in *Acetobacter* (see *Acetobacter* section below). pMV24 was used to study the regulation and function of the genes *ginI*, *ginA* and *ginR* of a quorum-sensing system from a *K. intermedius* strain producing three different *N*-acylhomoserine lactones (Iida et al. 2008a). The data demonstrated that the GinI/GinR quorum-sensing system controls the expression of *ginA*, which in turn inhibits oxidative fermentation, including acetic acid and gluconic acid fermentation by an unknown mechanism. In a further study, it was discovered that expression of the outer membrane protein gene *gmpA* is positively controlled by the GinA protein in an unknown manner (Iida et al. 2008b). Complementation studies with the expression of *gmpA* using pMV24 demonstrated that GmpA plays a role in inhibiting the formation of oxidized products, including acetic acid and gluconic acid. Transcriptome analysis revealed *gltA* encoding a putative glycosyltransferase, *pdeA* encoding a putative cyclic-di-GMP phosphodiesterase, and *nagA* encoding a putative N-acetylglucosamine-6-phosphate deacetylase as further target genes whose expression is influenced by the GinA protein. For functional and phenotypic analysis *gltA* and *nagA* were expressed under the control of both their own promoter as well as the promoter P_*lac*_ of pMV24 and *pdeA* was expressed from P_*lac*_ in *K. intermedius* (Iida et al. 2009). Recently, pMV24 was used to express the acyl-CoA dehydrogenase (ACAD) gene *caiA* from a thermotolerant *K. intermedius* in a non-thermotolerant *K. medellinensis* strain (Konjanda et al. 2019). It improved growth, acetic acid tolerance and ethanol oxidation even at higher temperature.

### Other pBR322-based expression plasmids used in *Komagataeibacter*

Another pBR322-based plasmid is pUCD2 originally developed for *Agrobacterium* and exhibiting autonomous replication and an active P_*tet*_ promoter of the *tetC* gene for tetracycline resistance in *K. hansenii* (Deng et al. 2013). pUCD2 was used to test the complementation of *K. hansenii* Cel^-^ mutants by expressing the cellulose synthase complex gene *acsA*, the guanylate dicyclase gene *dgc1*, and the putative transcriptional regulator gene *crp*-*fnr* (*ccp*) from their native promoters (Deng et al. 2013). In this study, pUCD2 with a promoterless *tetC* gene was also used to test promoter activities in *E. coli* and *K. hansenii* to study the *acs* operon and *ccp* promoter regions. pUCD2 was also used for complementation by expressing lysine decarboxylase and alanine racemase genes fused at the 3’ ends (C-terminally) with the sequence for an octapeptide FLAG epitope tag allowing detection via immunoblotting (Deng et al. 2015). Furthermore, pBR322 was used to create the shuttle vectors pBE2 and pBE3 by ligating linearized native pGE2 and pGE3 plasmids from *K. europaeus* with pBR322 (Akasaka et al. 2015). pBE2 and pBE3 can replicate in *K. europaeus*, but were not used further. Another pBR322-based vector is pCTP1 containing a chlamydial plasmid cloned into pBR322 and used to express *acsD* of the cellulose synthesizing operon *acs* in fusion with *gfp* from P_*lac*_ to produce N-terminal and C-terminal fusion proteins in complementation studies of a *K. xylinus acsD* disruption mutant (Mehta et al. 2015). The data obtained suggested that the AcsD protein aids in the proper orientation of the linear terminal complexes along the longitudinal axis of the cell, thus AcsD is involved in the final stage of the hierarchical assembly of cellulose.

### pSEVA plasmids and regulatable expression in *Komagataeibacte*r

The most recent lineage of expression plasmids used in *Komagateibacter* is based on the *Standard European Vector Architecture* (SEVA) toolkit, a resource for constructing optimal plasmid vectors based on a minimized backbone and three interchangeable modules to design a compilation from several origins of replication, diverse antibiotic resistance markers, and a cargo of interest, flanked by uncommon restriction sites (Durante-Rodriguez et al. 2014). Based on SEVA, a complete set of tools was developed for engineering *Komagataeibacter* that consists of protocols, modular plasmids, promoters, reporter proteins, and inducible constructs that should enable external control of gene expression (Florea et al. 2016a). Eight SEVA-based plasmids with different *ori* and low, medium or high expected copy number were assessed for propagation in *K. rhaeticus* iGEM. With *ori* RK2, pBBR1, and RSF1010 also mentioned above or below and pWV01, the respective SEVA plasmids were found to show replication, while with *ori* R6K, pRO1600/ColE1, pMB1 and ColE1/pBR322 the SEVA constructs tested did not show replication. The latter, *ori* pBR322 could be used in *Komagataeibacter* at least in other plasmids as mentioned above. From seven reporter proteins tested, mRFP1, GFPmut3, and sfGFP showed visually detectable expression. With mRFP1 as reporter 10 promoters from an open-access collection of synthetic minimal *E. coli* promoters were tested for their promoter strength in *K. rhaeticus* iGEM. For inducible gene expression, four constructs expected to be induced externally by anhydrotetracycline (ATc) or *N*-acyl homoserine lactone (AHL) were tested. From these, the AHL-inducible constructs showed higher induction and lower leakiness than the ATc-induced constructs, yet the induction fold-changes were only up to 5-fold under the conditions tested (Florea et al. 2016a). Interestingly, the LuxR-dependent AHL-inducible system showed a much better induction performance due to a very low basal mRFP1 signal when non-induced and a very strong mRFP1 signal when cells were induced inside cellulose pellicles. Furthermore, the *E. coli* Hfq protein that binds small regulatory RNAs (sRNAs) and mRNAs to facilitate mRNA translational regulation and a sRNA targeting UDP-glucose pyrophosphorylase (UGPase) mRNA were co-expressed from a SEVA plasmid with pBBR1 *ori* in response to the AHL inducer to inhibit UGPase mRNA translation. UGPase catalyzes the production of UDP-glucose, the substrate for cellulose synthesis, and is encoded by a single gene in the genome, allowing knockdown by a single sRNA. This system was found to be highly efficient, as cellulose production was suppressed completely upon full induction and could be fine-tuned using different concentrations of AHL. With *E. coli* Hfq and broad-host-range backbone, the system was engineered to be a general platform for targeted knockdowns in *Komagataeibacter* and other bacterial species independent from the host Hfq by adding new sRNA sequences to the plasmid making the construct easily modifiable for other mRNA targets (Florea et al. 2016a).

The pWV01 *ori* found to be functional in the above study with a SEVA backbone is also present in the non-SEVA plasmid backbone pBAV1C containing the l-arabinose-inducible *araC*-P_*araBAD*_ system, which was used to induce expression of the *bcs* operon genes *bcsA*, *bcsAB*, and *bcsABCD* in *K. xylinus* for engineering and characterization of bacterial nanocellulose films as low cost and flexible sensor material (Mangayil et al. 2017). Despite several attempts, the pBAV1C derivative with pWV01 *ori* could not be isolated from the transformed *K. xylinus* cells suggesting instability, although clear phenotypic differences were detected in growth curves and cellulose production in the presence of the plasmid.

The SEVA-based *luxR*-P_*lux*_ system mentioned above was extended by including the AHL-synthesis gene *luxI* downstream of the synthetic constitutive promoter J23104 on a separate plasmid to establish synthetic cell-to-cell communication in *K. rhaeticus* (Walker et al. 2019). Expression of *luxI* allowed the production of the diffusible AHL molecule N-(3-oxohexanoyl) homoserine lactone in the transformed *K. rhaeticus* strain (sender). In another *K. rhaeticus* strain carrying the *luxR*-P_*lux*_ system (receiver), the signal molecule was sensed, affecting the expression of the *mRFP* gene encoding the fluorescence reporter protein. It was demonstrated that communication can occur both within and between growing pellicles in mixed cultures of the two strains.

SEVA-based plasmids were also used to characterize 11 constitutive promoters, 3 inducible promoters (P_*lux*_, P_*tet*_, P_*araBAD*_), natural and synthetic terminators and ribosome binding sites as well as protein degradation tags in *K. xylinus*, *K. hansenii*, and *K. rhaeticus* iGEM (Teh et al. 2019). P_*lux*_ was found to be stronger and less leaky than P_*tet*_ and P_*araBAD*_. Due to the high leakiness, induction fold changes of P_*tet*_ and P_*araBAD*_ were rather small and in the case of P_*araBAD*_ depended on the carbon source supplemented for growth. In this study, CRISPR interference (CRISPRi) with a catalytically inactive *Streptococcus pyogenes* Cas9 protein (dCas9) was also tested as a tool to readily knock down the expression of a target gene by blocking transcription. Therefore, the dCas9 gene fused to a 3xFLAG tag DNA sequence was expressed by the strong promoter J23104 together with a single guide RNA (sgRNA) gene under the control of the native tracrRNA promoter from *S. pyogenes*. Alternatively, the sgRNA gene was positioned without promoter just downstream of the *dCas9*-3xFLAG gene, thus forming an operon. The mentioned constructs were cloned into a single pSEVA331Bb plasmid backbone. When targeting the 5’-ends of the *acsAB* gene and of the *acsD* gene of the endogenous *acs* operon for cellulose synthesis in *K. hansenii* by two different sgRNAs, a more than 2-fold decrease in *acsAB* expression and 15% reduced cellulose yield were observed, while for *acsD* no significant change in expression was observed.

pSEVA-based CRISPRi was also used in *K. xylinus* to control the expression level of *galU* encoding UGPase controlling the metabolic carbon flux between the cellulose synthesis pathway and the pentose phosphate pathway (Huang et al. 2020). By overexpression of *galU* and interfering with different sites of the *galU* sequence using CRISPRi, varying expression levels of *galU* from 3 to 3000% were obtained. Analysis of the cellulose characteristics showed that porosity was negatively and crystallinity was positively correlated with increasing *galU* expression levels. This results also confirmed that the properties of the bacterial cellulose can be altered without chemical modifications to increase the application potential in different fields. Furthermore, these studies showed that CRISPRi as well as Hfq-mediated RNA interference can be used to modulate target gene expression in *Komagataeibacter* and potentially also in other AAB.

To enhance bacterial cellulose production by *K. xylinus*, the effect of the expression levels of the UGPase gene *galU*, the phosphoglucomutase gene *pgm*, and the nucleoside-diphosphate kinase gene *ndp* were analyzed using pSEVA331 derivatives and synthetic RBSs exhibiting different strength identified via fluorescence-activated cell sorting (FACS) in a GFP reporter library (Hur et al. 2020). With pSEVA331-based expression of all mentioned genes under the control of P_J23104_ and with a selected synthetic RBS, the bacterial cellulose titer was 4-fold higher under shaking conditions (3.7 g/L) than that of wild-type *K. xylinus*. In static conditions 5.3 g/L cellulose was reached, demonstrating that reinforced metabolic flux toward bacterial cellulose through modified gene expression represents a practical approach for the improvement of bacterial cellulose production. By another strategy, which included the deletion of a PQQ-dependent glucose dehydrogenase gene and co-expression of the glucose facilitator gene *glf* from *Zymomonas mobilis* and the endogenous glucokinase gene *glk* from *K. xylinus*, a somewhat higher cellulose titer (5.9 g/L) was obtained with *K. xylinus* in a specific growth condition (Liu et al. 2020b). In this study, expression plasmid pRedGX with a pBBR1 *ori*, a kanamycin resistance gene *kanR*, *lacI*^q^, and the λ-*red* gene controlled by LacI^q^-dependent P_*trc*_ was constructed via Gibson assembly in a SEVA-like manner. This plasmid was used for the IPTG-induced expression of λ-Red recombinase gene in order to increase the efficiency of homologous recombination between the targeted chromosomal gene-flanking regions and a linear PCR product consisting of the in-frame deleted gene and chloromycetin resistance gene with flanking FLP recognition target (FRT) sites, all flanked by the targeted gene-flanking regions. A second expression plasmid, pFLPGX, containing *lacI*^q^, a pBBR1 *ori* flanked by FRT sites, a spectinomycin resistance gene, and the FLP recombinase gene *flp* controlled by LacI^q^-dependent P_*trc*_, was also constructed via Gibson assembly. pFLPGX with the same *ori* as pRedGX but a different resistance gene was used to eliminate the λ-*red* plasmid by antibiotics selection and to produce FLP recombinase, which catalyzes the reciprocal recombination between the FRT sites introduced into the chromosome for in-frame gene deletion and also between the FRT sites in the *flp* plasmid (FRT-pBBR1 *ori*-FRT) for self-elimination of the *flp* plasmid. Using the λ-*red* and the *flp* expression plasmids, four putative glucose dehydrogenase genes were deleted in *K. xylinus*, resulting in the identification of one of these genes as being responsible for gluconate formation from glucose. In the respective mutant, the *glf* and *glk* genes were constitutively co-expressed from a plasmid with a pBBR1 *ori* and a kanamycin resistance gene. The results obtained indicated that glucose was transported into the cell by the facilitator protein and glucokinase further increased the production of cellulose (Liu et al. 2020b). In contrast, the constitutive expression of the glucose phosphotransferase system genes *ptsG* and *ptsHIcrr* from *E. coli* did not significantly increase the efficiency of glucose utilization, likely because of limited availability of PEP, which is the donor of the phosphate group during PTS-catalyzed glucose uptake.

### Genomic integrations for target gene expression in *Komagataeibacter*

In two studies, genomic integrations instead of plasmids have been used for the expression of target genes in *Komagataeibacter*. A wild-type *K. xylinus* strain was modified by random transposon mutagenesis to insert the *E. coli* β-galactosidase gene *lacZ* generating a lactose-utilizing and cellulose-producing mutant strain (Battad-Bernardo et al. 2004). The promoterless *lacZ* gene expressed from the Tn*10* cassette inserted once into the chromosome was constitutively expressed, alleviated the growth retardation in lactose medium and was stably maintained in a non-selective medium for more than 60 generations. The modified strain showed a 28-fold increase in cellulose production when grown in lactose medium and could utilize 17 g/L of lactose in whey substrate within 4 days. In a genomic and metabolic analysis of a *K. xylinus* strain producing bacterial cellulose nanofiber (CNF), glucose-6-phosphate isomerase and 6-phosphogluconate dehydrogenase encoded by *pgi* and *gnd* have been predicted as novel overexpression targets for the enhanced CNF production (Jang et al. 2019). Therefore, the heterologous *pgi* and *gnd* genes from *E. coli* and *Corynebacterium glutamicum* were individually constitutively overexpressed from the chromosomal *sacB* locus under the control of P_*tac*_. By this approach, the amount of CNF produced in a complex medium containing glucose was doubled compared to that obtained with the control strain.

## Target gene expression in *Acetobacter*

For *Acetobacter*, we found 30 publications reporting on recombinant DNA work with expression plasmids and 2 major lineages can be seen. The first studies were published in 1985 by Teruhiko Beppu and co-workers. Here, the first chimeric shuttle vectors for *E. coli* / *A. aceti* (*Acetobacter* subgenus *Acetobacter*) have been constructed by ligation of cryptic plasmid pMV102 endogenously present in *A. aceti* subsp. *xylinum* NBI 1002 with the *E. coli* plasmids pACYC177, pBR322, or pBR325. The initial size of chimeric plasmids (>6 kb) was reduced by a series of consecutive digestion and ligation steps delivering pMV329 with only 3.4 kb as the smallest vector (Fukaya et al. 1985a). Most of the constructed shuttle vectors were stably maintained in *Acetobacter*. In parallel, improvements in the chemically induced competence for the transformation of *Acetobacter* were reported (Fukaya et al. 1985c; Okumura et al. 1985). Furthermore, shuttle vectors pMVL1 and pMVL2 were constructed by ligation of the pMV102 plasmid with a pBR322 derivative containing the β-isopropylmalate dehydrogenase gene *leuB* from *A. aceti* under control of its native promoter (Okumura et al. 1988). Therefore, pMVL1 and pMVL2 carrying an ampicillin resistance cassette were the first *A. aceti* / *E. coli* shuttle vectors with double selection markers as the *leuA* gene allowed selection of *leu*^+^ transformants in an *A. aceti leuA*^*-*^ host. Both pMVL1 and pMVL2 appeared to be stably maintained in *A. aceti* as plasmids after 4 times of successive inoculation and cultivation.

### pMV24—the first major expression plasmid used in *Acetobacter*

The smallest plasmid of the pMV series described above (pMV329; 3.4 kb) was used to construct the shuttle vector pMV24 by inserting pMV329 between the *Aat*II and *Nde*I sites of pUC18 (Fukaya et al. 1989). Plasmid pMV24 exhibits an estimated copy number of 10 in *A. pasteurianus*, promoter P_*lac*_, and allows translational fusion of the target protein with the 10 N-terminal amino acids of the *E. coli* β-galactosidase. pMV24 was used for recombinant DNA work in 13 *Acetobacter* studies for expression of various genes from the P_*lac*_ promoter present on the plasmid and additionally from the native gene promoter, if present on the cloned insert:

(i) A membrane-bound aldehyde dehydrogenase ALDH from *A. polyoxogenes* fused to the short N-terminal β-galactosidase peptide was produced in an *A. aceti* mutant lacking the enzyme activity (Fukaya et al. 1989). In submerged fermentation, expression of the ALDH-encoding gene caused an approximately 2-fold increase of the production rate and the maximum concentration of acetic acid.
(ii) Complementation studies with acetic acid-sensitive mutants of *A. aceti* obtained by chemical mutagenesis revealed three genes termed *aarA*, *aarB*, and *aarC* responsible for acetic acid resistance. Gene *aarA* was identified to encode a citrate synthase (Fukaya et al. 1990).
(iii) The *aarC* gene expressed from the pMV24 backbone was found to functionally complement an *aarC* gene disruptant mutant of *A. aceti*, which is defective in acetate assimilation (Fukaya et al. 1993).
(iv) A part of the promoter region of the membrane-bound alcohol dehydrogenase (ADH) gene of *A. pasteurianus* was cloned into pMV24 to corroborate a second transcriptional start by primer extension analysis (Takemura et al. 1993b). Furthermore, a promoterless chloramphenicol acetyltransferase (CAT) gene was cloned into pMV24 resulting in pMVC18 usable for promoter analysis by CAT activity assays. It was shown that the more than 10-fold increased ADH activity caused by ethanol was not due to increased transcription of the *adh* gene, suggesting a mechanism involving translational or posttranslational regulation.
(v) In a search for genes conferring ethanol resistance, an ethanol-sensitive *A. pasteurianus* mutant was transformed with a pMV24-based genomic library and the recombinants were screened for ethanol resistance. The *his1* gene encoding histidinol phosphate aminotransferase was shown to be responsible for the suppression of the ethanol sensitivity of the mutant. This effect was dependent on the higher copy number of *his1* obtained with plasmid pMV24, as the single genomic *his1* copy was not sufficient to enable ethanol resistance (Takemura et al. 1993a).
(vi) The *adhS* gene encoding subunit III of the three-component membrane-bound alcohol dehydrogenase (ADH) from *A. pasteurianus* was expressed from pMV24 and restored the ADH activity of an *adhS* mutant deficient in subunit III (Kondo et al. 1995).
(vii) In a follow-up study, the *gdhS* gene for subunit III of the ADH from *G. suboxydans*, which strongly differs in its amino acid sequence from GdhS of *A. pasteurianus*, was expressed in the *A. pasteurianus adhS* mutant and could not restore the ADH activity (Kondo and Horinouchi 1997). (viii) A study on the esterase-encoding genes *est1*, which is induced only in the presence of ethanol, and *est2*, which is repressed in the presence of ethanol, suggested a *cis*-acting domain exerting transcriptional regulation in the 5’-region of *est2* mRNA (Kashima et al. 1999). Furthermore, the *est1* promoter activity was analyzed using the CAT reporter plasmid pMVC18 introduced above to demonstrate the in vivo effect of ethanol on the transcription of *est1*.
(viii) In a follow-up study, *est1* was shown to play a major role in ethyl acetate formation and its production was fully recovered in an *A. pasteurianus est1* mutant by expressing an *est1*-containing DNA fragment from pMV24 (Kashima et al. 2000).
(ix) In *A. aceti*, pMV24-based overexpression of *groESL* encoding the chaperones GroEL and GroES, which are induced not only by temperature shifts but also by other stresses such as exposure to ethanol, conferred improved growth of *A. aceti* at increased temperature (42°C) and also supported the resistance against ethanol stress (Okamoto-Kainuma et al. 2002).
(x) Similarly, pMV24-based overexpression of the molecular chaperone operon *dnaKJ* with its heat-shock promoter resulted in improved growth of *A. aceti* compared to the control strain at high temperature or in the presence of ethanol, suggesting a correlation to resistance against various stressors present during fermentation, although it did not increase the resistance to acetic acid (Okamoto-Kainuma et al. 2004).
(xi) Acetic acid resistance of *A. aceti* was increased when the aconitase gene was overexpressed from P_*lac*_ using pMV24, showing that multiple copies of the aconitase gene, and accordingly an increase in aconitase activity, conferred acetic acid resistance (Nakano et al. 2004).
(xii) In *A. pasteurianus*, pMV24-based overexpression of the *uvrA* gene, whose product is involved in recognition and processing of DNA lesions, resulted in increased biomass formation in the presence of high acetic acid concentrations and resulted in upregulation of enzymes involved in ethanol oxidation and acetic acid tolerance. UvrA thus contributes to acetic acid tolerance by protecting genome integrity, thereby ensuring gene expression and metabolism (Zheng et al. 2018).

### pCM62—the second major expression plasmid used in *Acetobacter*

In other studies with *Acetobacter*, the improved broad-host-range cloning vector pCM62 was used as an expression plasmid. It was originally developed for use in methylotrophs and other Gram-negative bacteria by combining the functions present in the minimal transferable and selectable RK2 replicon of the pTJS75 derivative pCM51 with the polylinker and pBR322 *ori* of pUC19 (Marx and Lidstrom 2001). pCM62 was used to express from the promoter P_*lac*_ or the native promoter, if present on the inserted fragment, a number of different genes:

(i) The UDP-galactose synthesis gene *galE* was expressed for complementation of a *galE* mutant of *A. tropicalis*, which was found to produce extracellular polysaccharide secreted into the medium instead of forming capsular polysaccharide involved in pellicle formation (Deeraksa et al. 2006).
(ii) The subunit III gene *adhS* of the PQQ-dependent alcohol dehydrogenase (ADH) from *A. pasteurianus* was expressed to complement an *adhS* disruptant mutant, which revealed that subunit III is essential for the formation of the active ADH complex besides subunit I and II (Masud et al. 2010).
(iii) Several selected genes identified to support growth of *A. tropicalis* at higher temperatures were expressed to complement gene disruptant mutants obtained by transposon mutagenesis (Soemphol et al. 2011). The study revealed a serine protease, glutamine synthetase, lysyl-tRNA synthetase, 3-phosphoglycerate dehydrogenase, and a few other proteins to rescue the growth defect of the respective mutant strain at higher temperature (42°C).
(iv) The chaperone genes *groESL* were expressed in an *A. pasteurianus groEL* disruptant mutant for complementation and in the parental strain, which enhanced the resistance against 4% acetic acid and 5% ethanol (Theeragool et al. 2018).
(v) The membrane-bound aldehyde dehydrogenase genes *aldFGH*, which are indispensable for acetic acid formation from ethanol, and the isozyme genes *aldSLC*, whose mRNA levels were 10-fold lower during growth on ethanol compared to growth without ethanol, were expressed in *A. pasteurianus* (Yakushi et al. 2018a).
(vi) A mutated oxidoreductase gene *fabG* that may change the fatty acid composition and supports growth of *A. pasteurianus* in a low-nutrient environment were expressed. It enhanced growth of *A. pasteurianus* and the acetic acid production useful for cost-effective manufacturing of rice vinegar (Phathanathavorn et al. 2019).

### Other expression plasmids used in *Acetobacter*

Other shuttle vectors for cloning were prepared using the replication region of the large native ~19 kb plasmid pAC1 from *A. pasteurianus* and the kanamycin or tetracycline resistance markers obtained from pUC4-KAPA and pBR322 fragments to yield pACK1 (4.5 kb), pACT7 (3.9 kb) and its smaller *Bal*I / *Pvu*II-digested and self-ligated derivative pACT71 (3.3 kb) (Grones and Turna 1992). Plasmid pACT7 was further modified to include a kanamycin resistance cassette enabling double selection, resulting in plasmid pACG3 (4.8 kb). These shuttle vectors for *E. coli* / *A. pasteurianus* exhibit only the replication region from pAC1, yet possess a similar ability to replicate in *E. coli* and *A. pasteurianus* as the shuttle vectors of the pMV series developed for *A. aceti* mentioned above. The plasmids with pAC1 replicon only were successfully expressed in twelve *Acetobacter* species and transfer of plasmid DNA was optimized for *A. pasteurianus* (Bilska and Grones 2003; Grones and Turna 1995). The pAC1 replicon only plasmid pACT71 was used to express the *E. coli* β-galactosidase gene from P_*lac*_ in *A. pasteurianus* to analyze its production and release of the protein through the outer membrane in dependence of the medium composition (Grones and Bencova 1994).

In a study on the functions of the multiple heme *c* moieties in intramolecular electron transport and ubiquinone reduction by ADH, the expression plasmid pKS13 providing tetracycline resistance was used to express the alcohol dehydrogenase (ADH) genes *adhAB* from *A. aceti* in *A. pasteurianus* NP 2503 deficient in ADH (Matsushita et al. 1996). Later, the two cloning and expression vectors pMK10 and pMK20 were constructed from the small *A. pasteurianus* plasmid pAP1 (3 kb) conferring kanamycin resistance, which can also replicate in *E. coli* (Kretova and Grones 2006). Plasmid pMK10 was constructed from a pAP1 fragment containing the origin and the *Hae*II fragment of pUC19 carrying the *lacZ*’ gene. Plasmid pMK20 was constructed from another pAP1 fragment with the origin fused with the *Eco*RI-*Pst*I fragment of plasmid pCE30 carrying the P_L_ and P_R_ promoters and the gene encoding the temperature-sensitive repressor *cI*^857^ from bacteriophage lambda. The plasmids pMK10 and pMK20 were stable 96–98% both in *A. pasteurianus* and *E. coli* after 250 generations in the absence of kanamycin. Plasmid pMK20 was successfully used for cloning and expression of a membrane protein from *Salmonella* (unpublished), but no further work based on these plasmids was reported.

In one *Acetobacter* study, the IPTG-inducible P_T7_-based Novagen vector pET-30a(+) with pBR322 origin was described to have been used in *A. pasteurianus* for inducible expression of the endogenous genes of DnaA- and DnaB-like proteins as well as the *dnaA* and *dnaB* genes from *E. coli* to study the replacement of the corresponding endogenous proteins expressed from the chromosome (Bugala et al. 2016). A strong induction of the plasmid-based gene expression in *A. orleanensis* was reported, yet it was not explained whether it resulted from the T7 promoter present on pET-30a(+) or from another promoter included by the cloning procedure. Furthermore, plasmid backbone pBAD18 carrying pBR322 origin, the classical l-arabinose-dependent AraC regulator gene *araC* and the P_*araBAD*_ target promoter of AraC have been used in *A. orleanensis* for arabinose-inducible expression of asRNA fragments (121 nucleotides) which are antisense to the C-terminal coding sequence of the *dnaA* and *dnaB* gene sequences studied (Bugala et al. 2016). The data of the asRNA induction by arabinose have not been shown, thus a conclusion about leakiness and induction performance is not possible yet.

In two recent *Acetobacter* studies, the pBBR1-based broad-host-range cloning vectors pBBR1MCS-4 and pBBR1MCS-2 already introduced above in the *Gluconobacter* section were used. pBBR1MCS-4 was used to express in *A. pasteurianus* the *adhAB* operon of the endogenous PQQ-dependent alcohol dehydrogenase (ADH) from its native promoter P_*adh*_ (Wu et al. 2017). By this, the acetic acid production was improved while the residual ethanol content was decreased. 2D proteomic analysis indicated that 19 proteins with several functional classifications were differently expressed more than 2-fold upon *adhAB* overexpression. Metabolic flux analysis of the pathway from ethanol and glucose revealed that overproduction of the PQQ-dependent ADH is an effective way to improve the ethanol-oxidizing respiratory chain. pBBR1MCS-2 was used in *A. pasteurianus* to express genes of the membrane-bound alcohol dehydrogenase (*adhA*) and aldehyde dehydrogenase (*aldH*), PQQ biosynthesis genes (*pqqAB* or *pqqABCDE*), and combinations thereof, each under the control of the promoter P_*tuf*_ that was 1.8-fold stronger than P_*adhA*_ in GFP reporter assays (Gao et al. 2020a). Synergistic expression of these genes could not only efficiently relieve the conflict between increased acetic acid production (69 g/L in semi-continuous cultivation) and compromised cell fitness, but also enhanced the acetic acid tolerance of *A. pasteurianus* to a high initial concentration of 3% (v/v) thereby shortening the duration of the starting-up process from 116 to 99 h. This strategy is of significance for decreasing costs for producing high-strength acetic acid industrially and will also be useful for the production of other desired organic acids, especially those involving PQQ-dependent enzymes.

## Target gene expression in *Gluconacetobacter*

### Plasmid diversity in *Gluconacetobacter*

Taking into account the AAB genera and strain updates, as well as our exclusion criteria mentioned before, we found 10 studies using diverse plasmids for gene expression in *Ga. diazotrophicus*. Mostly, electroporation was used to transfer the recombinant DNA, yet conjugation was also used in some cases. The first recombinant DNA study in *Gluconacetobacter* was a complementation experiment in a levan synthesis-deficient mutant of the renamed *A. diazotrophicus* (Arrieta et al. 1996). Here, the plasmid pPW12, a derivative of the broad-host-range cosmid cloning vector pLAFR1 conferring tetracycline resistance and derived from pRK290 with RK2 *ori*, was used to construct a cosmid library from total genomic DNA of the parental strain to express endogenous complementing genes from their native promoters. This approach revealed a gene termed *lsdA* encoding a levansucrase potentially interesting for the production of the trisaccharide kestose from sucrose. The isolated complementing cosmid termed p21R1 was mobilized into the parental strain by conjugal mating to increase the gene copy number and thereby the levansucrase yield, finally resulting in 189 units mL^-1^ representing more than 95% of the total proteins secreted under selected culture conditions (Hernandez et al. 1999). In a further study, this levansucrase and its D309N derivative were enzymatically characterized after cloning their genes into broad-host-range cloning vector pRK293 and expression from the native *lsdA* promoter (Batista et al. 1999). A later study demonstrated the importance of *lsdA* for adaptation to high osmotic stress and for biofilm formation (Velazquez-Hernandez et al. 2011). The *lsdA* mutant showed a decreased tolerance to 50–150 mM NaCl, a 99% reduced tolerance to desiccation, and a decrease in the ability to form cell aggregates on abiotic surfaces. Complementation of the mutant by expressing *lsdA* again from its native promoter, both cloned as a fragment into broad-host-range plasmid pBBR1MCS-3 (see *Gluconobacter* section), recovered the abilities of the parental strain in the *lsdA* disruptant mutant.

*Ga. diazotrophicus* as an endophytic nitrogen-fixing plant-associated microbe is generally also interesting as a host to express genes encoding proteins that could enable control of pests attacking agricultural plants. In this context, the *Bacillus thuringiensis cry* genes are interesting since they encode δ-endotoxins exhibiting entomopathogenic activity. To test the control of coleopteran pests in sugarcane, the *cry3A* gene hooked in the transposon Tn*5* was introduced into *Ga. diazotrophicus* by conjugation on the narrow-host-range suicide plasmid backbone pSUP1021 carrying the transfer origin *oriT* of plasmid RP4 and integrating into the genome (Salles et al. 2000). The *cry3A* gene was successfully expressed in nitrogen-fixing conditions when fused to the promoter of the *nifHDK* operon from *Rhizobium leguminosarum* biovar *trifolii*. The use of regulated promoters activated only under specific conditions may avoid the problem of insect resistance caused by constant production of the δ-endotoxin. Later, the *Cry1Ac* gene from *B. thuringiensis* var. *kurstaki* was introduced into *Ga. diazotrophicus* after cloning into the double replicon shuttle vector pKT230 (Subashini et al. 2011). Clones containing the *Cry1Ac* gene produced the 130 kDa toxin protein both when present inside the sugarcane tissue as well as *ex planta*, and it did not impair the process of nitrogen fixation. To evaluate the colonization process of sugarcane plantlets and hydroponically grown rice seedlings by *Ga. diazotrophicus*, strain PAL5 was marked with *gusA* encoding β-glucuronidase and *gfp* encoding green fluorescent protein (Rouws et al. 2010). The *gusA* and *gfp* reporter genes were constitutively expressed under the control of the gentamicin resistance gene promoter on the broad-host-range plasmid backbone pBBR1. The reporter plasmids were shown to be valid tools to detect strain PAL5 and monitor colonization.

Recently, pBBR1MCS-5 (see *Gluconobacter* section) containing the mCherry gene under control of the promoter P_tac_ was used to analyze the colonization of *Ga. diazotrophicus* on two genotypes of elephant grass (*Pennisetum purpureum*), a perennial C4-plant employed for grazing, silage production or bioenergy, via seed coat and leaf spray (Camelo et al. 2020). The results showed that one elephant grass genotype produced a higher amount of biomass and total nitrogen than the other when *Ga. diazotrophicus* was sprayed on leaves. When colonizing plants and performing nitrogen fixation, the limited availability of iron might impose restrictions on the expression, assembly, and function of the nitrogenase complex. To better understand the iron uptake mechanisms in *Ga. diazotrophicus*, an ATP-binding cassette (ABC) transport system comprising the three genes *feuABC* encoding a periplasmic-binding protein, a permease, and a traffic ATPase was analyzed earlier (Urzua et al. 2013). In this study, the *feuABC* operon was expressed from its native promoter on a pJB_3_Tc_20_-based plasmid backbone with the RK2 replicon in a *Ga. diazotrophicus* parental strain and its *feuAB* disruptant mutant under iron-replete and iron-depleted conditions. The complementation of the mutant with the plasmid carrying the *feuABC* operon restored growth under iron-restricted conditions to the level seen for the parental strain, indicating that *feuABC* is needed for iron uptake.

### Promoter screening in *Ga. diazotrophicus*

For *Ga. diazotrophicus*, a promoter library was constructed using the promoter probe vector pPW452, a derivative of the broad-host-range vector pMP220 obtained from pTJS75 with an RK2 replicon and containing a multiple cloning site upstream of a promoterless *lacZ* reporter gene (Schwab et al. 2016). With the library prepared from total *Ga. diazotrophicus* DNA, 480 clones were screened and six promoters were isolated and characterized further. They showed variable expression strengths in the presence of organic acids and polyalcohols, or glucose and sugarcane juices. The characterized promoters might be used in the future for the expression of genes of interest in *Ga. diazotrophicus*. Interestingly, three out of the six characterized promoters are probably involved in the transcription of antisense RNA, including the one showing the highest expression strength. However, a tight and strongly conditionally regulated promoter was not revealed in this study.

## Target gene expression in *Acidomonas*

As described before, the AAB genus *Acidomonas* was established since several characteristics of the former species *Acetobacter methanolicus* could be distinguished from other type and representative AAB including *Acetobacter* strains, resulting in the renaming of *A. methanolicus* to *Acidomonas methanolica* (Urakami et al. 1989). For *Acidomonas* expression vectors including a basic promoter probe vector have been created based on the popular broad-host-range multi-copy plasmid RSF1010 (Schröder et al. 1989; Schröder et al. 1991). In *A. methanolica*, a RSF1010-based derivative was used to express the core antigen gene of hepatitis B virus subtype ayw under control of the *E. coli* P_*lac*_ promoter alone and under tandem control of the strong promoter P_*acm*_ from the *Acetobacter* phage *Acm*l and from P_*lac*_. When produced from the tandem promoters, the core antigen detected in lysates of *A. methanolica* was about 7-fold higher than in *E. coli* (Schröder et al. 1991).

The P_*acm*_ promoter and the *E. coli* promoters P_*lac*_ and the temperature-dependent phage lambda P_R_ promoter were tested in a RSF1010-based vector for expression of the pectate lyase gene *ptlB* from *Erwinia carotovora* and the aspartokinase/homoserine dehydrogenase genes *thrAB* from *E. coli* in *A. methanolica* (Föllner et al. 1994). The highest specific activities of the enzymes were detected with P_R_ in tandem arrangement with *ptlB* and its native promoter and with P_*acm*_ in tandem with *thrAB* and its native promoter. A temperature shift to activate the P_r_ promoter was not needed in *A. methanolica* and was even disadvantageous, since it drastically lowered the resulting specific enzyme activity. The created vector system was also stable in *A. methanolica* in antibiotics-free media.

The RSF1010-based promoter probe vector pRS201 carrying a promoterless *lacZ* gene as a reporter was used to analyze the induction of the stationary growth phase promoters P_*bolA*P1_ and P_*fic*_ from *E. coli* in Gram-negative bacteria including *A. methanolica* (Miksch and Dobrowolski 1995). Transcriptional activation of P_*fic*_ in *A. methanolica* was growth phase-dependent as in *E. coli* and was increased 6- to 7-fold during the transition to the stationary phase. Subsequently, P_*fic*_ was used to test growth phase-dependent expression and production of a secreted hybrid β-glucanase consisting of 107 amino-terminal residues of *Bacillus amyloliquefaciens* β-glucanase and 107 carboxy-terminal residues of *B. macerans* β-glucanase in *A. methanolica* using a Tn*5*-based transposon cassette non-specifically integrating into the chromosome (Miksch et al. 1997). Due to P_*fic*_, the β-glucanase was highly overexpressed and secreted into the medium during the stationary phase, while total and extracellular production of β-glucanase varied depending on the transposon integration site. The viability of the bacterial cells was not affected and cell lysis did not occur.

## Target gene expression in *Acidiphilium*

The transfer of genetic information into *Acidiphilium* and the construction of suitable vectors started about 30 years ago (Roberto et al. 1991). Plasmids from different incompatibility groups have been tested to determine the frequency with which they are transferred from *E. coli* to *Acidiphilium* strains. During these studies, a conjugative function has been discovered in *Acidiphilium* strains by observing the transfer of mobilizable, but not self-transmissible plasmids between *Acidiphilium* cells (Roberto et al. 1991). In following studies, DNA transfer into *Acidiphilium* by conjugation but also by electroporation was established and improved (Glenn et al. 1992; Inagaki et al. 1993). The pRK2 replicon-based broad-host-range plasmids pRK415 and pLAFR3 were used to analyze transformation efficiencies, which turned out to depend on the recipient strains (Glenn et al. 1992). Also, the *E. coli* / *Acidiphilium* shuttle vector pAH101 was constructed by fusing a restriction fragment from the native pAH1 plasmid from *Acidiphilium* sp. 42H with a fragment carrying the β-lactamase gene from pUC19 as resistance marker. Transformants of *A. facilis* could grow in ampicillin-containing medium only with pAH101, showing that the β-lactamase gene was efficiently expressed from the native Tn*3* promoter region also in *A. facilis* (Inagaki et al. 1993). However, the pAH101 plasmid appeared to be unstable in *A. facilis* in the stationary phase.

In further work, the plasmid pRK415 conferring tetracycline resistance was used to express the arsenic resistance genes *arsABC* from a natural *E. coli* isolate in *Acidiphilium* (Bruhn and Roberto 1993). After conjugal transfer of the constructed plasmid pIRC107 from *E. coli* to *Acidiphilium*, the plasmid was stable for at least one week with and without tetracycline selection. Also, the transformed *Acidiphilium* strain survived longer and in higher numbers in cultures with arsenopyrite ore than the parental strain did, suggesting a functional expression of the *arsABC* operon from its native *E. coli* promoter. Genetically improved strains of acidophilic bacteria were considered to have the potential to optimize microbial leaching of metals from ores. Enhancing their resistance to toxic metals is one option for strain improvement.

*Acidiphilium* could also be used to degrade organic pollutants in acidic wastewaters, provided they harbor the required genes. To evaluate the potential of *Acidiphilium* for biodegradation in acidic environments, gene transfer systems were tested by using the IncP1 antibiotic resistance plasmids RP4 and pVK101 and the phenol degradation-encoding plasmid pPGH11 (Quentmeier and Friedrich 1994). The plasmids RP4 from *Pseudomonas*, pVK101 (RK2 and pHK17, see *Gluconobacter* section for pVK) and pPGH11 (R68.45 and pPGH1 from *Pseudomonas*) could be transferred into *A. cryptum* with frequencies of 1.8 x 10^-2^ to 9.8 x 10^-4^ transconjugants per recipient cell. In the transconjugants, the antibiotic resistances and the ability to degrade phenol were expressed. *A. cryptum* with pPGH11 grew with 2.5 mM phenol at a doubling time of 12 h and a yield of 0.52 g dry cell weight per g of phenol.

In a later study, the native pAM5 plasmid from *A. multivorum* was sequenced and analyzed to use its characteristics for the construction of a shuttle vector (Singh and Banerjee 2007). The plasmid pSK2 containing the pAM5 *rep* region, the pBR322 *ori* and the ampicillin resistance gene *ampR* from pUC19, and a multiple cloning site, was able to replicate and stably maintained in *E. coli*, *Acidiphilium* as well as in *Acidocella*. The plasmid copy number of pAM5 and pSK2 in *A. multivorum* was determined to be 50-60. Apparently, no further study was published yet demonstrating the functional expression of target genes in *Acidiphilium* strains with pSK2.

## Target gene expression in *Asaia*

Representatives from AAB genera have been demonstrated to be naturally associated not only with plants (e.g., *Acetobacter*, *Asaia*, *Gluconacetobacter*, *Gluconobacter*), but also with insects. The presence of AAB genera in insects (e.g., the fruit fly *Drosophila melanogaster* and the honeybee *Apis mellifera*), that typically use plant materials and sugar-rich matrices as food sources, is seen as roles of these AAB in exploitation of the food. *Asaia* was also found in the malaria mosquito vector *Anopheles stephensi* and is also present in and cross-colonizing other sugar-feeding insects of phylogenetically distant genera and orders (Crotti et al. 2009; Favia et al. 2007). In these studies, for tracking one *Asaia* strain was constructed by tagging it with a plasmid derivative of pHM2 carrying the pBR322 *ori*. The plasmid pHM2-Gfp was used to functionally express the green fluorescent protein Gfp under control of the neomycin phosphotransferase promoter P_*npt*_ *II* found to be active in a broader range of species. Cross-colonization patterns of the body of the mosquitos *An. stephensi, Aedes aegypti* and the leafhopper *Scaphoideus titanus* have been documented with *Asaia* strains labeled with a chromosome- and a plasmid-encoded fluorescent protein. Another *Asaia* strain was constructed by the insertion of a mini-Tn*5* gene cassette into the chromosome. This cassette contained the *dsRed* gene encoding a red fluorescent protein under control of the ribosomal promoter P_*rrnBP1*_ from *E. coli*. Fluorescence and confocal microscopy analysis showed that the labeled *Asaia* strains efficiently colonized guts, male and female reproductive systems and the salivary glands.

The ability to cross-colonize insects of phylogenetically distant orders is an important property for the development of symbiont-based control of different vector-borne diseases. To test the potential of such a control by activation of insect immunity, the pHM2 backbone was used in *Asaia* to express the *Wolbachia* surface protein (WSP) gene with its signal peptide sequence under the control of P_*npt*_ *II* that was derived from a *Wolbachia* strain isolated from the nematode *Dirofilaria immitis* (Epis et al. 2020). The recombinant strain *Asaia*^WSP^ induced the activation of the host immune response in *An. stephensi* and *Ae. aegypti* mosquitos and it inhibited the development of the heartworm parasite *D. immitis* in *Ae. aegypti*. The recombinant *Asaia*^WSP^ was also found to activate macrophages and *Leishmania* killing stronger than the empty vector control strain did (Varotto-Boccazzi et al. 2020). These results consolidated the immune-stimulating property of WSP and make *Asaia*^WSP^ worth of further investigations as a potential tool for the control of mosquito-borne diseases and for immune prophylaxis and therapy of leishmaniases and other diseases that could be inhibited by macrophage activation.

## Outlook

For AAB a diversity of expression plasmids has been developed and tested for expression of target genes and major plasmid lineages can be identified that are applied most often in AAB, although hitherto in only seven out of 49 AAB genera. Expression of target genes was achieved using the corresponding native promoter region or using a limited number of characterized promoters. Apparently, P_*lac*_ allowing LacI-dependent induction of expression in *E. coli* is the most used heterologous promoter for expression in AAB. Varying the length of the P_*lac*_ region can increase or decrease target gene expression in *Acetobacter* likely due to 5’-UTR effects in the mRNA (Tonouchi et al. 1998a). Only two studies reported to have used LacI-dependent IPTG-induced target gene expression (Bugala et al. 2016; Liu et al. 2020b). Only in one study the LacI-dependent IPTG-induced expression performance has been reported (Liu et al. 2020b). By using the fluorescence reporter gene *mRFP1*, the results showed that according to fluorescence microscope analysis, only a fraction of *K. xylinus* cells showed mRFP1 fluorescence; thus, cell heterogeneity was observed. Furthermore, only 3-fold induction by IPTG was observed from 0.1 mM to 0.5 mM reaching a saturation level of mRFP1 signals at 0.4 mM and 0.5 mM, while the 0 mM condition was not reported yet. Thus, the performance of IPTG-induced expression in AAB still remains to be solved or at least to be improved for tight and homogenous LacI-dependent target gene expression.

Due to a deficiency in regulatable promoters, almost only constitutive promoters have been used in AAB. The greatest variety of endogenous, heterologous, and synthetic promoters functional in AAB and exhibiting weak, moderate, strong, or very strong activity is known for *Gluconobacter* and *Komagataeibacter* (Florea et al. 2016a; Hölscher and Görisch 2006; Hu et al. 2015; Kallnik et al. 2010; Mientus et al. 2017; Shi et al. 2014; Teh et al. 2019; Yuan et al. 2016a). These data will be helpful to test and broaden the range of usable promoters in other AAB genera. For *Gluconobacter*, the highest change of a promoter activity according to plasmid-based LacZ activities was reported for the promoter P_*idh*_ of the inositol dehydrogenase gene GOX1857 from *G. oxydans* 621H (Mientus et al. 2017). Depending on the carbon source, P_*idh*_ activity was very low on mannitol and even lower on glucose, while P_*idh*_ activity on sorbitol was 100-fold higher than on glucose. This probably is the highest fold-change difference reported so far for *Gluconobacter* or AAB in general. However, the molecular mechanism of P_*idh*_ regulation and the transcriptional regulator(s) of P_*idh*_ involved need to be unraveled as well as the performance and the suitability of P_*idh*_ for use in a regulatable endogenous expression system. The *R. leguminosarum* promoter of the *nifHDK* operon, which is activated under nitrogen-fixing conditions, was functional in plant-associated *Ga. diazotrophicus in planta*. Therefore, P_*nifHDK*_ might also be used in nitrogen-fixing AAB *ex planta* to control gene expression in dependence of the available nitrogen sources (Salles et al. 2000). The basal expression (tightness), induction performance and relative induced expression strength of the heterologous P_*nifHDK*_ promoter applied in AAB were not reported yet. Likewise, endogenous *nif* promoters from nitrogen-fixing AAB strains could be characterized and, if suitable, used to construct regulatable expression systems in nitrogen-fixing AAB. The system(s) could possibly be transferred with all regulatory requirements to non-nitrogen-fixing AAB.

Until now, for AAB only two well-documented inducible heterologous expression systems are available for only two species from different genera. As described above, one is based on an AHL-inducible *luxR*-P_*lux*_ system and the other one is a well-tunable l-arabinose-inducible *araC*-P_*araBAD*_ system with up to 480-fold induction (Florea et al. 2016a; Fricke et al. 2020). The AHL-inducible *luxR*-P_*lux*_ system was reported to enable strong induction in *Komagataeibacter*, yet the strength of the induction appeared to depend on the growth conditions. Induction ranged from only up to 5-fold due to high leakiness in the absence of the inducer to a very much better induction performance in cells inside cellulose pellicles (Florea et al. 2016a). Understanding the reason for this difference in induction performance may help to further improve the *luxR*-P_*lux*_ system and possibly other heterologous expression systems (e.g., TetR) still leaky in *Komagataeibacter* for full functionality in any growth condition. In *Komagataeibacter*, an l-arabinose-inducible *araC*-P_*araBAD*_ system was also very leaky and thus induction fold-change was very low (Teh et al. 2019). In contrast, in *G. oxydans* 621H, the heterologous *araC*-P_*araBAD*_ system from *E. coli* MC4100 was very tight and tunable by l-arabinose concentrations up to 1% (w/v) (Fricke et al. 2020). However, in *G. oxydans* 621H, the l-arabinose oxidation activity is high and contributes to the strong acidification of the growth medium by forming arabinonic acid causing a severe loss of reporter activities, while the d-arabinose oxidation activity is low (Fricke et al. 2020; Mientus et al. 2017; Peters et al. 2017). Thus, using d-arabinose-responsive AraC derivatives could circumvent the pH issue of l-arabinose induction and might probably also result in increased sensitivity toward arabinose concentrations. Therefore, engineered AraC mutant proteins with altered binding pockets which activate transcription in response to d-arabinose and not in response to l-arabinose could be tested (Tang et al. 2008). The AraC protein was also engineered to specifically respond to triacetic acid lactone, vanillin and salicylic acid (Frei et al. 2018). The new phenolic-sensing variants of AraC showed responses of more than 100-fold over the background in *E. coli* and were highly specific toward their target compound. That illustrates the potential of the AraC transcriptional regulatory protein for molecular sensing, reporting and target gene expression, which could possibly also be achieved and used in AAB.

In summary, after 35 years of constitutive target gene expression in AAB, we now have the first regulatable expression systems for AAB in hand. Further promising candidates are in sight for both heterologous and endogenous regulatable expression systems and future studies certainly will reveal more regulated AAB promoters. Additionally, the flexible SEVA toolkit, used within the AAB hitherto only in *Komagataeibacter*, is expected to speed up expression system development and testing or screening of such systems in even more AAB genera.
